# Extracting Mechanistic Information from an Open Data Set for a Pharma-Relevant Suzuki–Miyaura Cross-Coupling Reaction

**DOI:** 10.1021/acs.oprd.5c00298

**Published:** 2026-03-06

**Authors:** Barnabas A. Franklin, Niall W. B. Donaldson, James D. Firth, Christopher S. Horbaczewskyj, Aaron Aspell, Theo Tanner, Adrian C. Whitwood, Julie Wilson, Jessica K. Hargreaves, Ian J. S. Fairlamb

**Affiliations:** † Department of Chemistry, 8748 University of York, Heslington, York, North Yorkshire YO10 5DD, U.K.; ‡ Department of Mathematics, 8748 University of York, Heslington, York, North Yorkshire YO105DD, U.K.

**Keywords:** catalysis, mechanism, cross-coupling, ligand effects, data science, open data

## Abstract

Open reaction data sets, particularly those gathered through high-throughput experimental (HTE) reaction screening campaigns, hold considerable value in downstream data analysis and reaction understanding. Data can be used to reveal important trends. Such open reaction data are increasingly being reported and deposited to appropriate databases, particularly for the most popular chemical reactions, e.g., Suzuki–Miyaura cross-coupling (SMCC) of organohalides and organoboron compounds, catalyzed by Pd and mediated by a suitable base. While there is considerable complexity associated with SMCC reactions, as informed by many independent mechanistic studies over the years, one can bring out essential trends through detailed data analysis. In this study, we have taken an open reaction data set reported by Pfizer and evaluated the properties of closely related substrates and the effect of the reaction solvent, base, and ligands. Through a detailed analysis of the reaction outcomes, we have focused on the occurrence of a common side-product in SMCCs, resulting from protodeborylation of the organoboron coupling component. There is considerable benefit in exploring the main cross-coupled product yield compared with the amount of protoborylated compound formed. In our analysis, we have delineated several key and interesting trends, which reveal value in evaluating side-product(s)/main product yields. A Shiny app has been developed for the rapid evaluation of the SMCC reaction data set.

## Graphical abstract

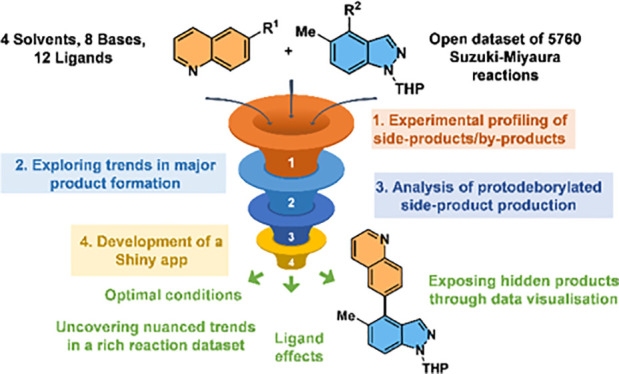

## Introduction

There is a high value associated with the knowledge of many outcomes for a given chemical reaction. High-throughput experimentation (HTE) allows for reaction variables (continuous and discrete variables) to be rigorously tested and analyzed downstream with appropriate data science tools.^1–4^ Indeed, it has been widely recognized that complex patterns of chemical reactivity can be delineated from organic reaction databases, which can ultimately inform mechanistic rationale and improved reaction conditions for a broader set of substrates.^5^

Recently, we have witnessed an increase in HTE data^6^ being made available as the Supporting Information in research publications and/or uploaded to appropriate open data repositories (e.g., University data repositories, GitHub, the Open Reaction Database).

An influential study was reported by a pharmaceutical-focused group from Pfizer, led by Richardson and Sach,^7^ which showcased the high throughput capabilities of a nanomole-scale flow reaction screening platform for a representative real-world example that required the use of the Suzuki–Miyaura cross-coupling (SMCC) reaction (Scheme 1). The study was unique in that several reaction variables (e.g., solvent, base, and exogenous ligand) and structural variations of the starting materials (in the organohalide/pseudohalide and organoboron reagents) were tested, with reaction outcomes spanning 0–100% conversion to cross-coupled product. Indeed, a total of ∼5760 reaction outcomes were reported in the Pfizer study, which were assayed by ultra performance liquid chromatography–mass spectrometry (UPLC-MS). Furthermore, data points were collected for reactions at a high rate (e.g., 1500 reactions every 24 h – each reaction is 1 min). As such, we recognized that this reaction outcome data set held valuable reaction outcome data. Within the Pfizer data set (Excel spreadsheet) reported,^7^ quantitative data (percentage conversions) for consumed starting material(s) and formed cross-coupled product were provided (determined by UV–vis detection in the UPLC, supported by confirmed MS information). Cronin and co-workers employed the use of this data to test the potential of their machine learning approach in the prediction of cross-coupling reaction yields,^8^ with good success, despite the hidden reaction complexities and differences associated with these catalytic reactions (e.g., catalyst speciation issues^9–15^ for different phosphines and no details about side-products^16^) *vide infra*.

**Scheme 1:**
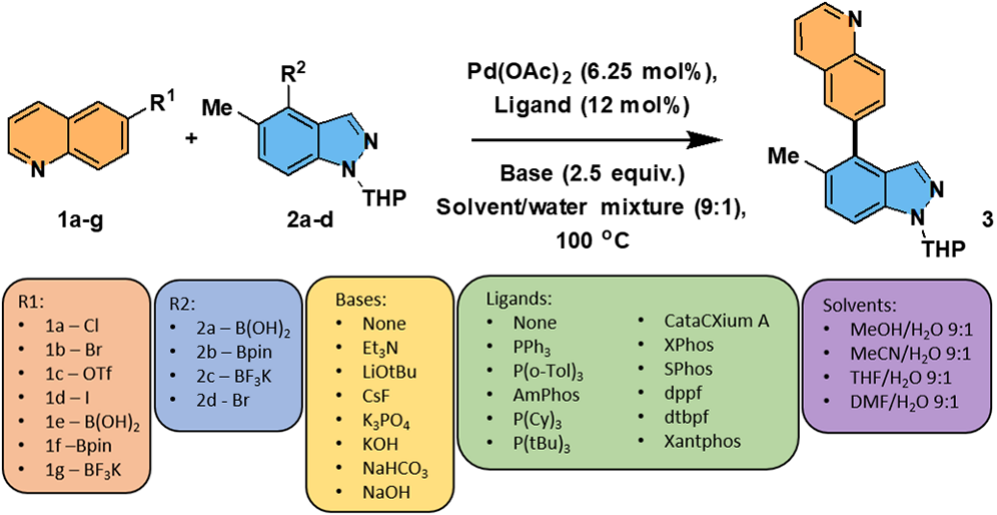
SMCC Reactions Examined in the Richardson–Sach Study^7^ ^a^

a Reaction cross-couples either the quinoline halide/pseudohalide/boron reagent (**1a**–**1g**) with the indazole halide/pseudohalide/boron reagent (**2a**–**2d**), varying base, ligand, and solvent combinations. The screen involved 11 ligands (plus one blank) × 7 bases (plus one blank) × 4 solvents.

We herein report a detailed investigation of the Pfizer SMCC reaction data set,^7^ employing appropriate data analysis tools to uncover important trends, limitations, and potential triggers for a dominant side-reaction, protodeborylation.

Our overarching goals of this study are outlined below:

1) Identify reactions and conditions that produce cross-coupled product **3** in good to high yields.
2) Reveal the underlying trends, particularly any solvent, base, and ligand effects.
3) Gain insight into reaction conditions that lead to higher quantities of side-products, revealing potential chemical sensitivities in the system.
4) Conduct an analysis of a complex data set for a topical reaction of wide academic and industrial interest.
5) Reveal whether there are any “needle in a haystack results” that might warrant further investigation, i.e., a simple phosphine ligand that performed surprisingly well.

## Results and Discussion

### Experimental Assessment of the Richardson–Sach SMCC Reaction^7^ and Profiling Side-Products/Byproducts

We began experimentally testing the SMCC reactions (outlined in Scheme 1) using batch reaction screening methodologies, including reactions conducted ‘one at a time’ in a standard round-bottomed flask and 12-position reaction carousel. It became apparent that side reactions were operating for the reactions tested in our hands, particularly in the presence of air. SMCC reactions can be complicated by protodeborylation^17^ and homocoupling^18^ side-reactions with respect to either or both coupling partners. Other competing side-reactions can involve hydrolysis of the organoboron species or protodehalogenation of the organohalide electrophile.^19^ This is readily exemplified by the reactions of quinoline halides **1a**–**1d** with boron-containing indazole derivatives **2a**–**2c,** which principally afford the cross-coupled product **3**, along with independently characterized side-products, e.g., formed by either homocoupling of both reagents, protodeborylation, or protodehalogenation (Scheme 2).

**Scheme 2:**
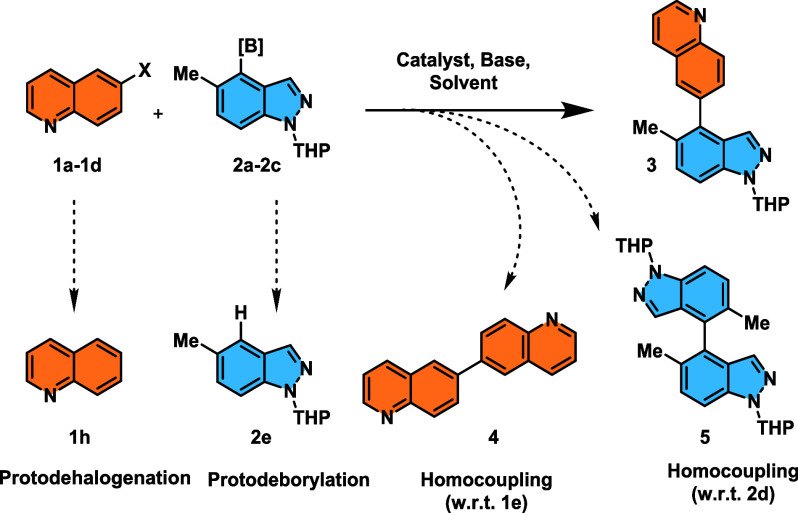
Reaction Details for the SMCC Reaction of Quinoline Halides **1a**–**1d** with Boron-Containing Derivatives Indazole **2a**–**2c**, Showing Formation of Cross-Product **3** and Side-Products **1h**, **2e**, **4,** and **5** ^a^

a We note that **5** could be a by-product of the Pd precatalyst activation step, as well as a side-product formed by oxidative homocoupling.

On realizing the complications associated with potential side-product formation, Sach and co-workers were able to kindly provide additional side-product information related to their original reaction screening data.^7^ Protodeborylation was noted as the major side-reaction. Thus, the revised data set contained critical information about starting material consumption, major product formation, and the dominant side-product (measured by UPLC-MS). With these data in hand, we recognized that a detailed evaluation of the reaction outcomes outlined in Scheme 1 could be conducted. Moreover, uncovering the potential triggers for protodeborylation could prove to be highly valuable, particularly for future HTE SMCC reaction screening campaigns.

### Preprocessing of Data

Prior to analysis, 268 observations were removed from the data set (see ESI for the data used: 5492 reaction outcomes), either due to inconsistencies within the data set or visible errors in a recorded variable. An additional variable was added, named “Missing Product”, which was calculated by 100 minus the mass balance of the reaction. This missing product is believed to consist of several unmeasured side-/byproducts, for example, quinoline **1h** and homocoupled products **4** and **5** (Figure 1) (i.e., most likely unidentified peaks from the original UPLC-MS analysis). The data were split into two main reaction groups, 1 and 2 (Figure 1). Group 1 contains the data where the boron species is on the indazole ring system, and Group 2 contains the data where the boron species is on the quinoline ring system. Groups 1 and 2 are therefore defined by a reversal of polarity, where the quinoline is the organohalide in Group 1 and the organoboron compound is the organoboron compound in Group 2 (Figure 1). Following analysis of these data, we concluded that the Group 1 reaction data set was of higher quality and value. As such, we placed our attention on Group 1 reaction data for the purposes of this study. Some analysis of the Group 2 reaction data can be found in the E.S.I.

**Figure 1:**
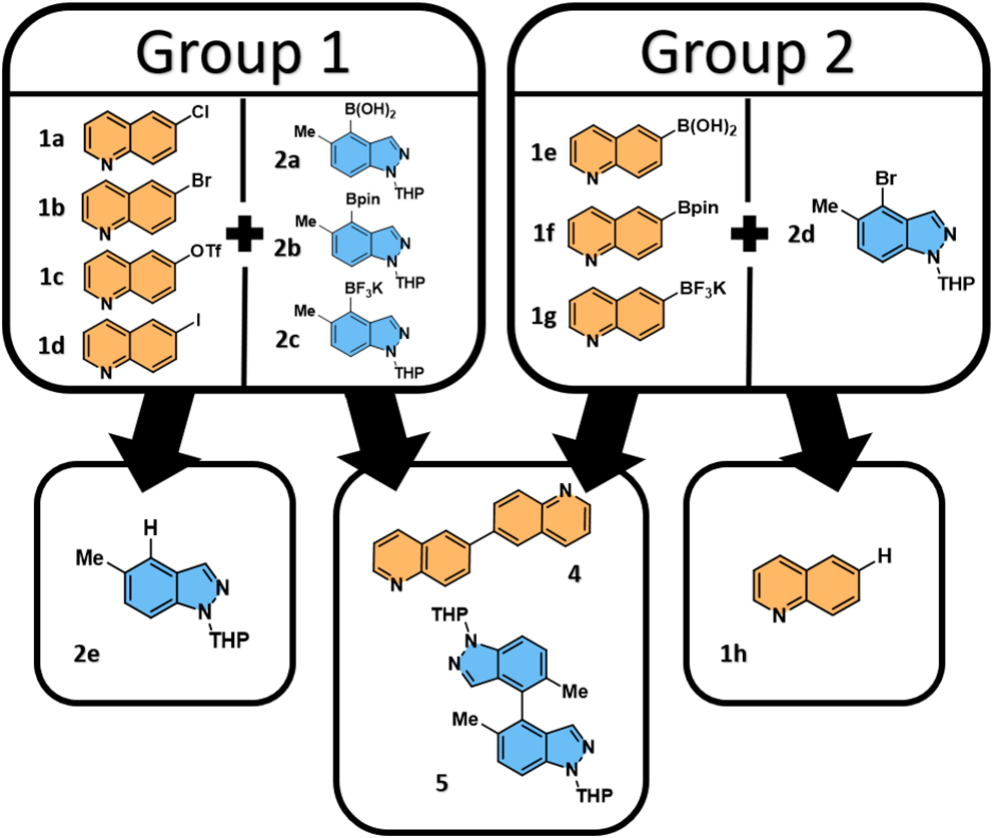
Representation of the different reactant combinations contained within groups 1 and 2, along with corresponding (potential) side-/byproducts, which can form depending on the identity of the organohalide and organoboron species used. A key was used in the original publication^7^ for compounds **1a**–**1d** (6-Cl-Q, **1a**; 6-Br-Q, **1b**; 6-OTf-Q, **1c**; 6-I-Q, **1d**) which has been maintained in the main text below to help the reader.

When the general reaction performance of each combination of electrophile and nucleophile is compared by product yield percentage, it is evident that BF_3_K (trifluoroborate) **2c** is the least reactive organoboron compound in this system (Figure 2). The vast majority of the SMCC reactions involving BF_3_K afforded <50% cross-coupled product **3**. Reactions that use either indazole boronic acid **2a** or boronic ester derivative **2b** perform significantly better (under the conditions tested), sharing similar reactivity. Irrespective of the choice of the organoboron derivative, there is a marked trend in (pseudo-) haloquinoline choice – the worst performing substrate being 6-Cl-Q **1a**, with its strong carbon-chloride bond (oxidative addition being more challenging^20,21^ ). The substrates, 6-Br-Q **1b** and 6-OTf-Q **1c**, perform similarly, whereas 6-I-Q **1d** is the most reactive substrate (based on overall product yield). While not surprising, this highly valuable data set confirms it.

**Figure 2:**
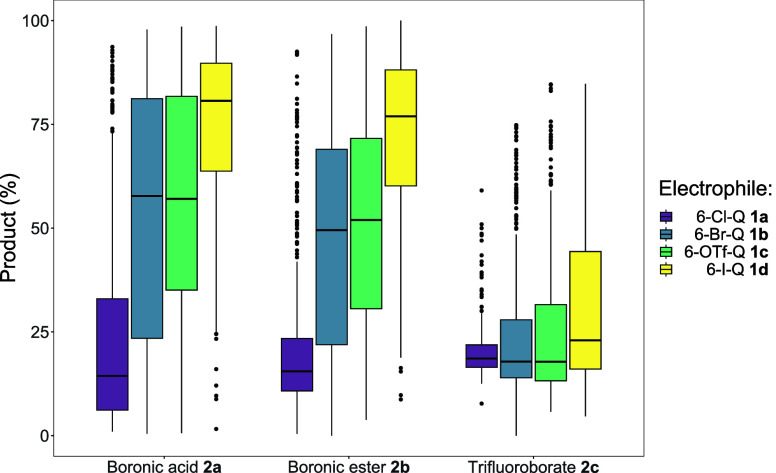
Boxplot for the Group 1 reactions, showing comparison of each combination of indazole boron species (**2a**–**2c**) with quinoline electrophile (**1a**–**1d**) with product (**3**) on the *y*-axis, indazole type on the *x*-axis and colored by quinoline type (note the coding “6-X-Q” was used in the original publication,^7^ referring to the type and position of the halogen (X) in the quinoline halide series **1a**–**1d**).

Moving further, cross-product **3** yields <35% are considered “low”, reactions in the range 35–65% are “medium”, and reactions above 65% are “high”. The structure of the halogenated quinoline highlights the reactivity differences (Figure 3). 6-Cl-Q **1a** exhibits lower reactivity, as indicated by the frequency of “low” product-yielding reactions. On the other hand, 6-I-Q **1d** is generally found in “high” yielding reactions. Higher cross-coupled product yields can therefore be attributed to weaker carbon–halogen bond strengths (i.e., a more efficient oxidative addition step). These correlations would be arguably obvious to the trained chemist, but perhaps less obvious to new postgraduate students, given the difference in carbon-halide bond strengths, with C–I being weaker than C–Cl. Clearly, other decisions (including waste factors, ease of synthesis, toxicity of the halide salt byproducts, use of low ppm Pd, and cost of the organohalide, i.e., ArI > ArBr ≫ ArCl)^22^ might drive whether an iodinated substrate is better or worse than a chlorinated substrate.

**Figure 3:**
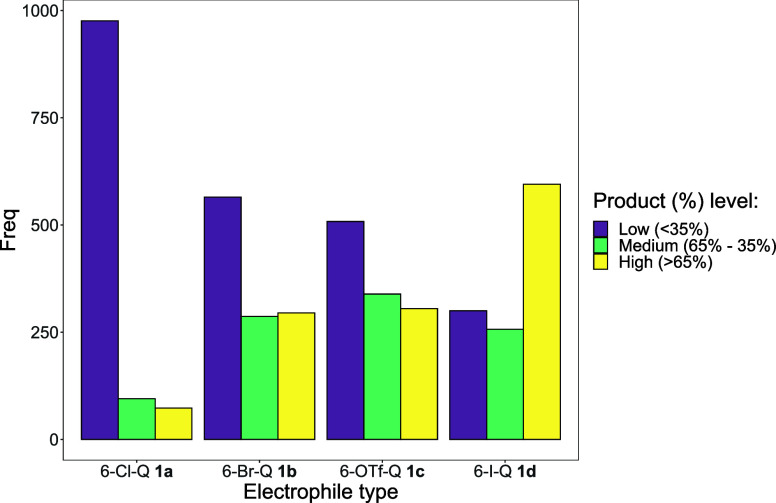
Bar chart frequency of each product yield level for each electrophile type (Group 1). Freq, frequency of reactions with a specific halogenated quinoline substrate.

To compare the most influential variables within Group 1, a series of plots was constructed. The stacked bar chart shown in Figure 4 (top) focuses on ligand type, for which there is significant variance in the number of “high” product level reactions. The ligand with the highest number is PPh_3_, with over half of the reactions affording “high” cross-coupled product yields. The worst ligand choice by some margin is Xantphos, which hinders the reaction. Xantphos is worse than using no exogenous phosphine ligand – a surprising outcome from this analysis, *vide infra*. Similarly, the next two worst-performing ligands are dppf and dbtpf, again, surprising, especially given how often they are used commercially, which highlights an incompatibility in this reaction system.^23^

**Figure 4:**
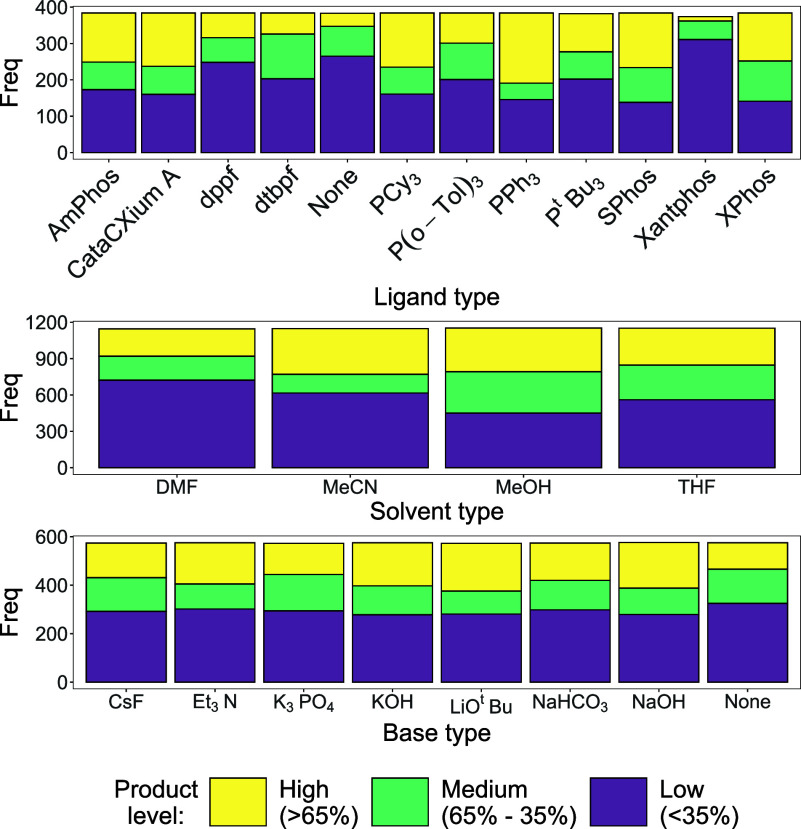
Three stacked bar charts. Top: Frequency of each product yield level for each ligand. Middle: Frequency of each product yield level for each solvent. Bottom: Frequency of each product yield level for each base.

The second stacked bar chart, shown in Figure 4 (middle), displays how the choice of solvent affects the product yield percentage for all observations in Group 1. When comparing solvents, there is an obvious worst performer – DMF. However, MeCN is arguably the best solvent, producing the largest number of “high” product level reactions, with MeOH a close second. The third stacked bar chart (Figure 4, bottom) compares the effect of base selection on the number of reactions at each product level. While different bases exert an effect, it is not as impactful as either ligand or solvent choice. Many of the bases perform similarly well, with LiO^*t*^Bu and NaOH being used in slightly more “high” product level reactions than the rest, and no exogenous base being involved in the least “high” product level reactions. By comparing these plots, the ligand choice affects the product yield percentage the greatest, spanning the best and worst performing reaction conditions. Ultimately, it is clear to see that all variables influence the reaction outcome significantly.

To further explore optimal reaction conditions, Group 1 was divided into the three separate organoboron compounds: indazole boronic acid **2a**; indazole boronic ester **2b,** and indazole trifluoroborate salt **2c**. A bubble count table is shown in Figure 5 for indazole boronic acid **2a**, illustrating reaction count by solvent type that are contained within each product yield level; the high-yielding reactions in the right-hand column are of particular interest. Acetonitrile (MeCN) is generally the best solvent as it has the most positive reaction outcomes in the “high” yield category.

**Figure 5:**
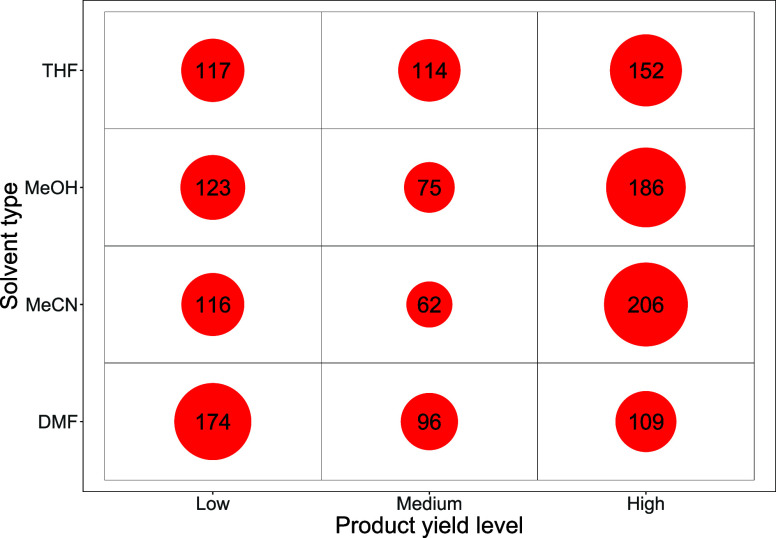
Bubble count table showing the count of reactions in each product yield level by solvent type (Group 1, using **2a** reaction data).

Each high-yield bubble was investigated further, and reactions were divided up by organohalide type (**1a**–**1d**), which is collated in Table 1.

**Table 1:** High-Yielding Reactions (for **3**) with Altering Organohalide Type (**1a**–**1d**) and Indazole Boronic Acid **2a** in Four Solvents

		solvent			
high-yielding reactions for product **3**		DMF	MeCN	MeOH	THF
quinoline electrophile	**1a**	6	14	0	24
	**1b**	20	45	56	47
	**1c**	22	63	45	28
	**1d**	61	84	85	53
	Total	109	206	186	152

Figure 6 illustrates the heatmap created by selecting boronic acid **2a** and 6-I-Q **1d** employing MeCN as the reaction solvent. This combination leaves only the ligand and the base available for a total of 96 reaction outcomes. Within this heatmap, 84 of the reactions can be considered “high” yielding. The reaction with the highest yield (98%) for **3** on this heatmap is PPh_3_, using LiO^*t*^Bu as the base (Figure 6). Several ligands perform well across the board, with PPh_3_, CataCXium A, and AmPhos being the best three. Reactions generally perform less well in the absence of an exogenous base. It is worth noting that in these systems, the coupling substrates can act as a base. The worst performing ligand is again Xantphos, a bidentate ligand that can be beneficial for many Pd-catalyzed cross-coupling reactions. However, Xantphos is known to be prone to side reactions, particularly aryl–aryl exchange between Pd^II^ and phosphorus.^24^

**Figure 6:**
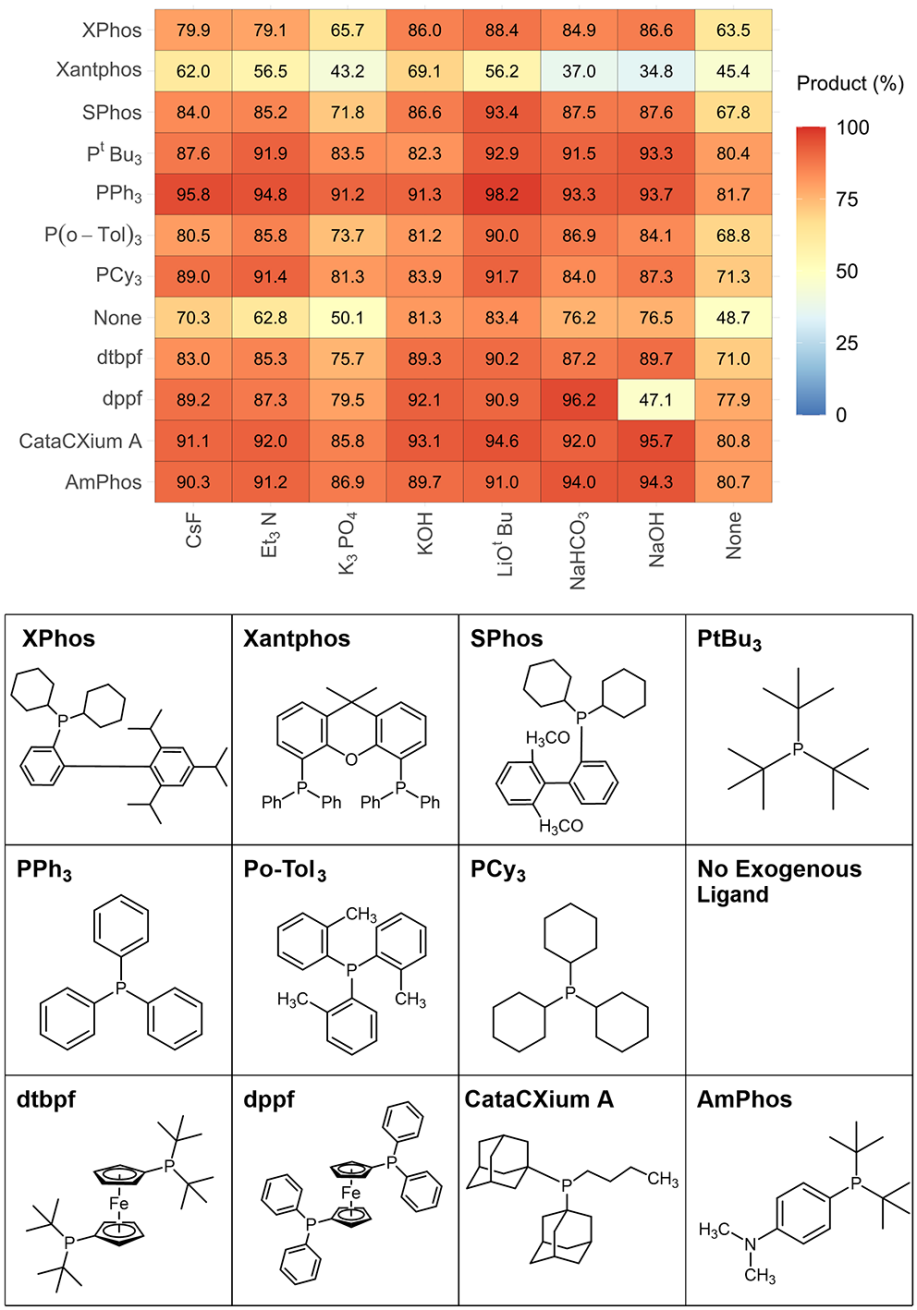
Heatmap for reaction combinations using indazole boronic acid **2a** and 6-I-Q **1d** (Group 1), using MeCN with varying ligand types with a heat scale spanning 0–100, where red represents a “high” yield, blue a “low” yield, and yellow an “intermediate” (middle-ground) yield.

As 6-I-Q **1d** has a high number of reactions in every solvent type (Table 1), heatmaps in a similar style to Figure 6 were also produced for MeOH, THF, and DMF (Figure 7). Here, the heatmaps show the best selected ligand and base combinations when using indazole boronic acid **2a** and 6-I-Q **1d** as the coupling partners. Figure 7 reveals MeCN as arguably the best solvent, with MeOH being a close second. The impact of the solvent-type relative to these other discrete variables is apparent in these data.

**Figure 7:**
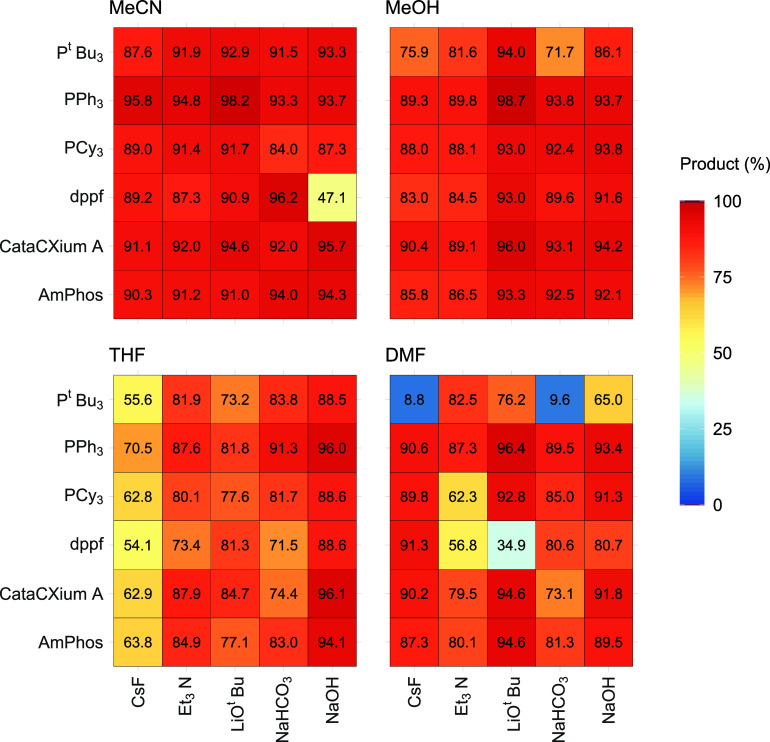
Four heatmaps for reaction combinations using indazole boronic acid **2a** and 6-I-Q **1d** (Group 1) with one for each solvent, using the six best selected ligands (rows) and five bases (columns) correlated with higher yields of product **3**.

### Analysis of Side-Product Formation (Protodeborylation)

We next switched our attention to the analysis of the side-product formation related to protodeborylation. Figure 8 shows the boxplots created when comparing the performance of each combination of organohalide and organoboron derivatives (in Group 1) by side product **2e** yield percentage. It is evident that most reactions employing the indazole trifluoroborate salt **2c** produce significant amounts of side product **2e** (many in the range >60%). Reactions that employ indazole boronic acid **2a** perform as expected with a large range of side product **2e** percentage, but with a clear pattern of decreasing order of organohalide reactivity (**1a** → **1d**).

**Figure 8:**
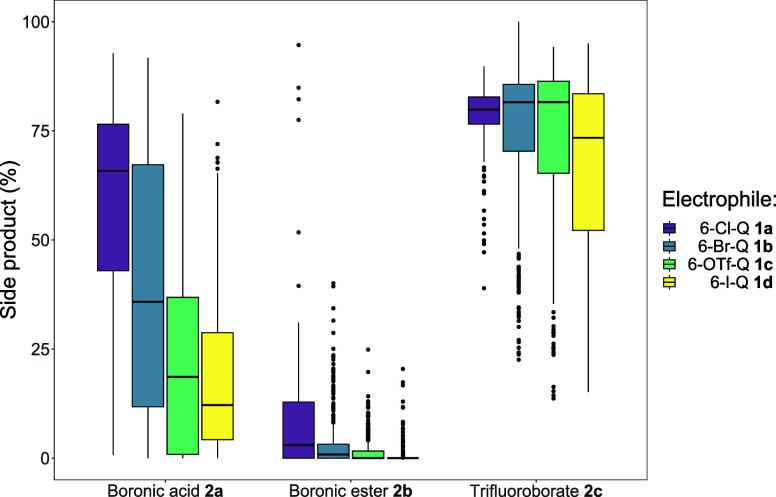
Boxplot for Group 1 reactions, showing comparison of each combination of indazole boron substrate **2a**–**2c** with quinoline halide **1a**–**1d**, correlated against the amount of side product **2e** formed on the *y*-axis, indazole type on the *x*-axis, and colored by quinoline type.

The reactions employing indazole boronic ester **2b** produce a comparatively low amount of side product with only six reactions >40%, but crucially, >50% of reactions producing no side product **2e**. This outcome has been further examined using our data analysis tool described later (the Shiny App *vide infra*).

Figure 9 illustrates a bubble count for reactions in Group 1 employing indazole boronic acid **2a**, highlighting side-product **2e** levels. The high-yielding side reactions as a function of organohalide type (**1a**–**1d**) and solvent are collated in Table 2. There is an uneven distribution of **2e** that forms from all of the quinoline halides **1a**–**1d**, with the number of high side product-producing reactions involving 6-Cl-Q **1a**, far higher than that of **1b**–**d**. However, in DMF solvent, the number of “high” side product-forming reactions employing 6-Br-Q **1b** is at a similar level to that of 6-Cl-Q **1a.**

**Figure 9:**
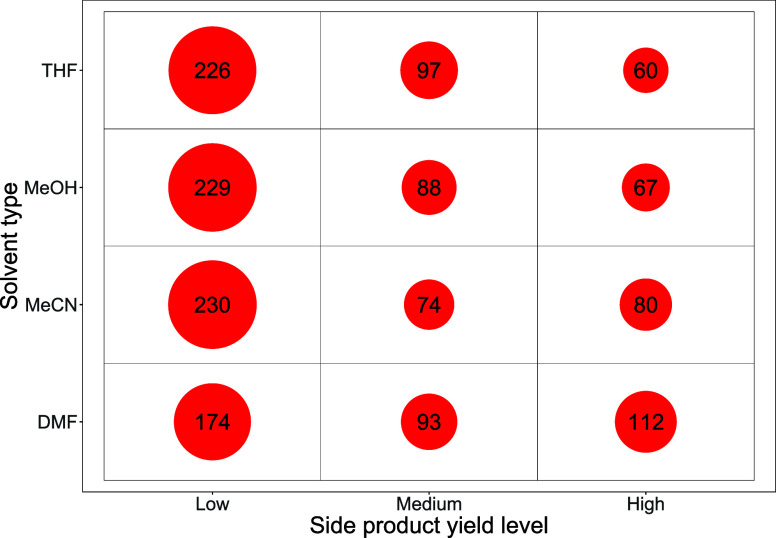
Bubble count table showing the count of reactions in each side-product yield level by solvent type for boronic acid **2a**.

**Table 2:** Reactions Affording Large Quantities of **2e**, with Altering Organohalide Type (**1a**–**1d**) and Indazole Boronic Acid **2a** in Four Solvents

		solvent			
high yielding reactions for side- product **2e**		DMF	MeCN	MeOH	THF
quinoline electrophile	**1a**	48	63	51	31
	**1b**	45	15	14	26
	**1c**	15	1	2	3
	**1d**	4	0	0	0
	Total	112	80	67	60

Figure 10 shows three heatmaps showcasing indazole boronic acid **2a** and 6-Cl-Q **1a** (Group 1). These heatmaps focus on six ligands and five exogenous bases, which contribute most to the protodeborylation side product **2e**. Note that there is some missing information for the Xantphos data using DMF as the reaction solvent (in the original data set^7^). Interestingly, the ligands PPh_3_ and CataCXium A also produced high quantities of protodeborylation side product **2e**, contrasting with the earlier analysis, which shows that the main product **3** can be formed in high yields using these ligands (cf. Figure 4).

**Figure 10:**
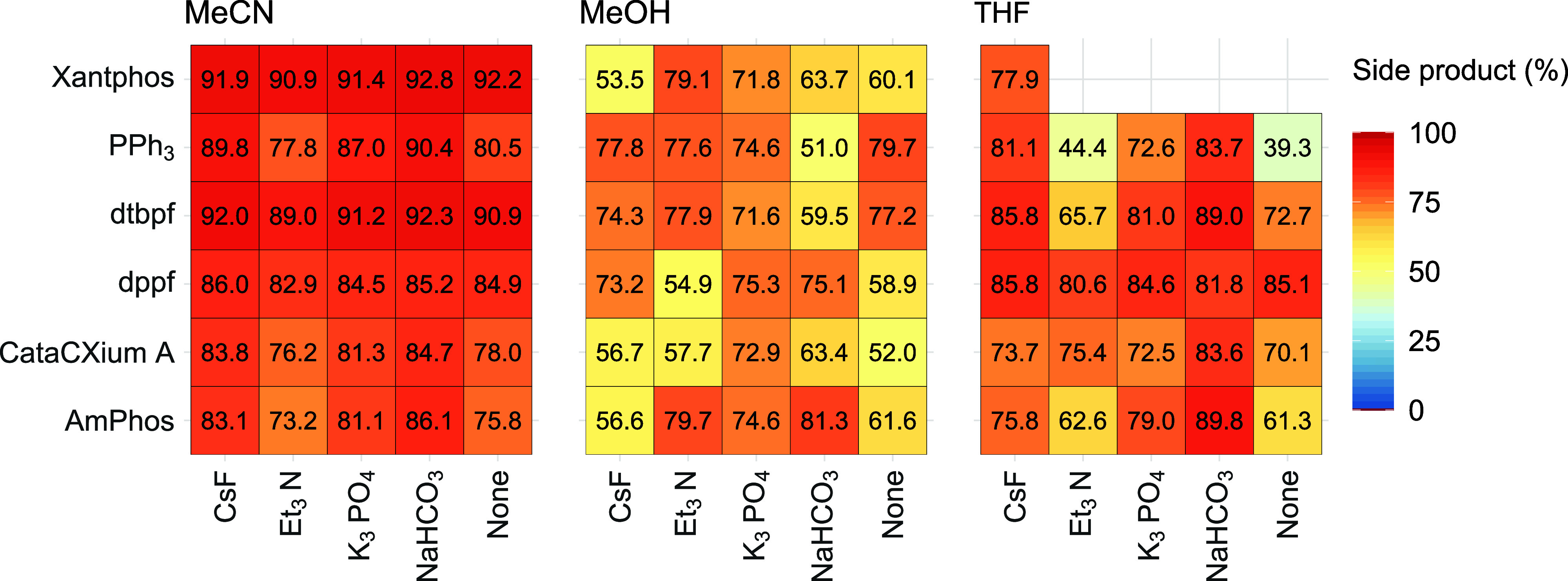
Three heatmaps for indazole boronic acid **2a** with 6-Cl-Q **1a** using the best selected six ligands (rows) and five bases (columns) leading to higher yields of side product **2e** (Group 1 reactions).

The next step was to examine the reactions that caused high quantities of protodeborylation side products to be formed by employing the indazole trifluoroborate salt **2c** substrate. Analysis was repeated in a similar fashion to above, using bubble count notation (Figure 11) and heatmaps (Figure 12).

**Figure 11:**
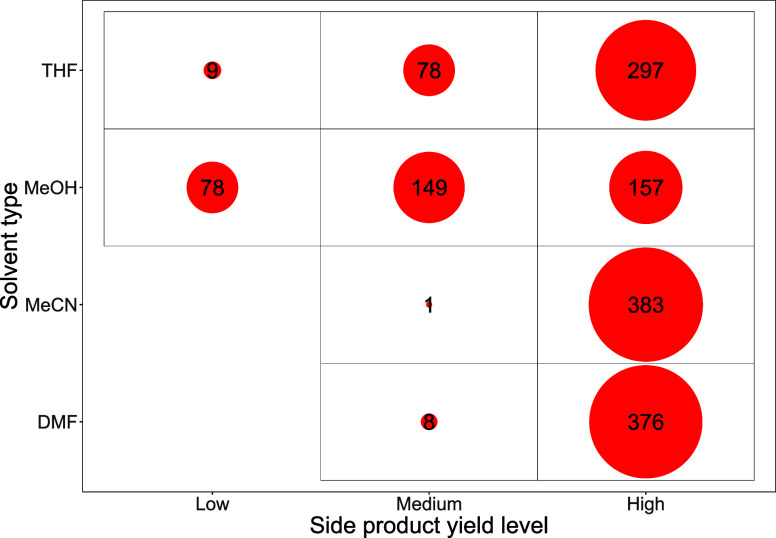
Bubble count table showing the count of reactions in each side product yield level by solvent type for BF_3_K derivative **2c** (Group 1 reactions).

**Figure 12:**
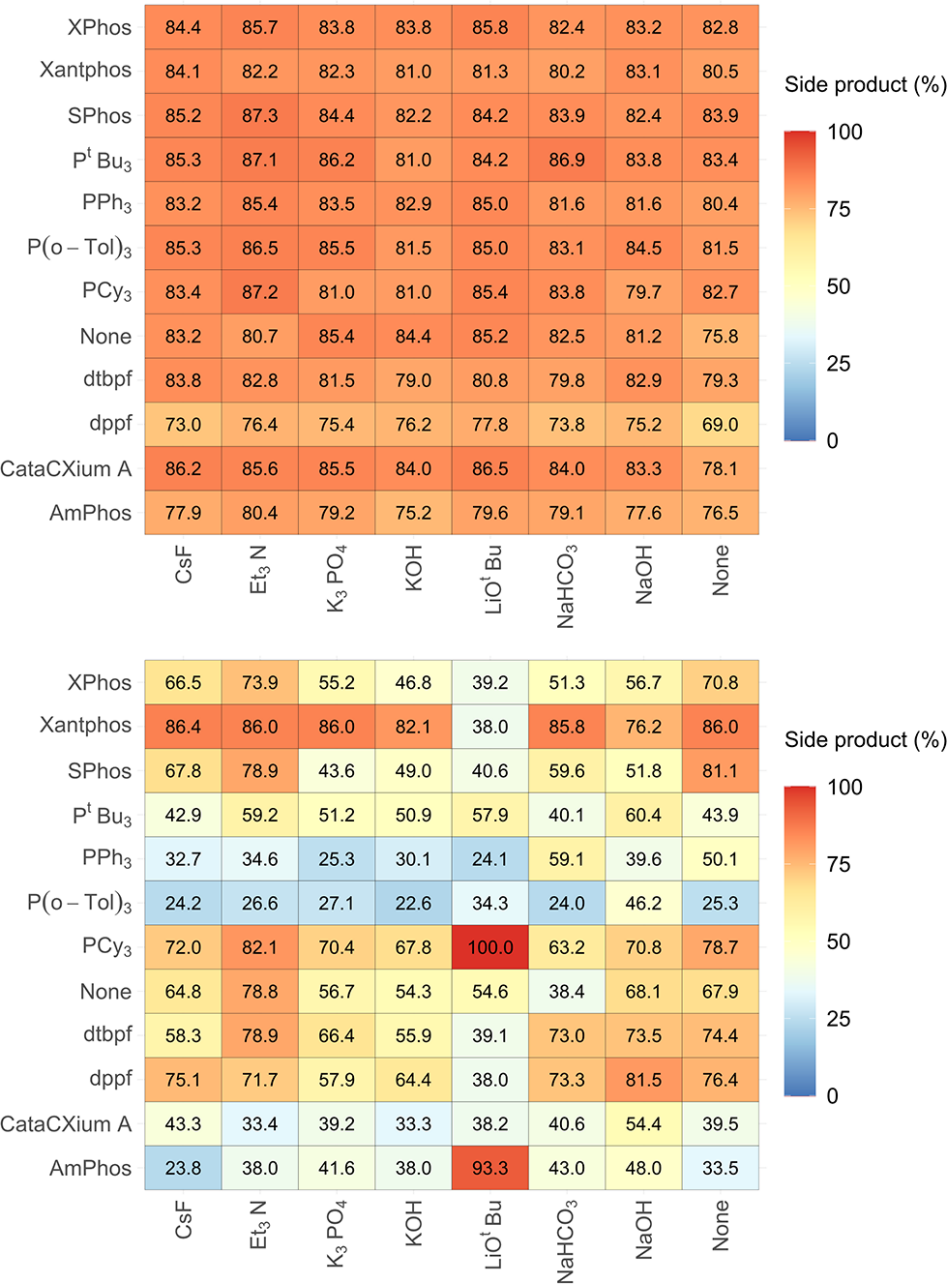
Two heatmaps for reaction combinations using BF_3_K **2c**. Top: 6-Cl-Q **1a** in MeCN solvent, Bottom: 6-Br-Q **1b** in MeOH solvent. Both plots have ligand type as the rows and base type as the columns with a heat scale ranging 0–100, where red is “high”, blue is “low”, and yellow is in the “mid-range”.

A large number of reactions involving indazole potassium trifluoroborate salt **2c** show that protodeborylation is a major competing side-reaction, forming **2e**, irrespective of the solvent type used (Figure 11). All but one reaction employing acetonitrile as solvent forms >65% **2e**. The SMCC reactions in methanol generally exhibit lower amounts of **2e**, *vide infra*. Dividing up the reactions by organohalide type (**1a**–**1d**) also reveals the extent of high side-product **2e** yielding reactions when **2c** was used (Table 3).

**Table 3:** Reactions Affording Large Quantities of **2e**, with Altering Organohalide Type (**1a**–**1d**) and Indazole Potassium Trifluoroborate Salt **2c**, across Four Solvents

		solvent			
high-yielding reactions for side-product **2e**		DMF	MeCN	MeOH	THF
Quinoline Electrophile	**1a**	94	96	84	97
	**1b**	96	96	34	80
	**1c**	93	96	32	70
	**1d**	93	95	7	52
	Total	376	383	153	297

Two heatmaps showing the distribution of **2e**, under these different reaction conditions and substrate identities, are shown in Figure 12. Irrespective of the type of activating phosphine ligand used, one can see the extent of protodeborylation in the data set using 6-Cl-Q **1a** and 6-Br-Q **1b** in acetonitrile and methanol solvents, respectively. Xantphos is generally a poor ligand in the reaction series employing 6-Br-Q **1b**. In one example using **1b** only **2e** is formed (when LiO^*t*^Bu was used as base and PCy_3_ was used as ligand). What is evident from these data is that there is significantly more ligand (catalyst) control in the reactions employing substrate 6-Br-Q **1b**.

### Development of a Shiny App

Shiny is a freely open-source web application framework for the program R. It allows users to build interactive, web-based data applications without needing extensive knowledge of web development.^25^ Shiny enables users to access dynamic and interactive tools for data analysis and visualization by combining the computational power of R with the accessibility of web applications. Shiny apps are popular in various fields, from academia and business analytics to healthcare and public policy. Shiny integrates with R packages like ggplot2, dplyr, and leaflet, which makes it straightforward to create complex visualizations or manipulate large data sets, as seen throughout this paper.

A Shiny app consists of two primary components: the user interface (UI) and the server function. The UI defines the layout and appearance of the app, and the server function contains the logic of the app, processing inputs from the UI and returning outputs in real time. This reactive framework allows Shiny apps to instantly reflect changes made by the user, whether they are filtering data, selecting variables, or modifying parameters. Shiny’s reactive programming model is key to its interactivity by continuously updating outputs based on user inputs without page reloading required.

The key advantage of a Shiny App is that it allows data scientists to share their insights with everyone, not just those who are fluent in R. This makes it an ideal tool for use in this research. Figure 13 displays an exemplar heatmap output created by using the Shiny app. The Shiny app can create four different types of plot: heatmap, boxplot, bar chart, and bubble chart for user inputted values such as group type or reactant type. This app bridges the gap between raw data and beautiful visualizations, giving users control over the data exploration process while providing a user-friendly interface.

**Figure 13:**
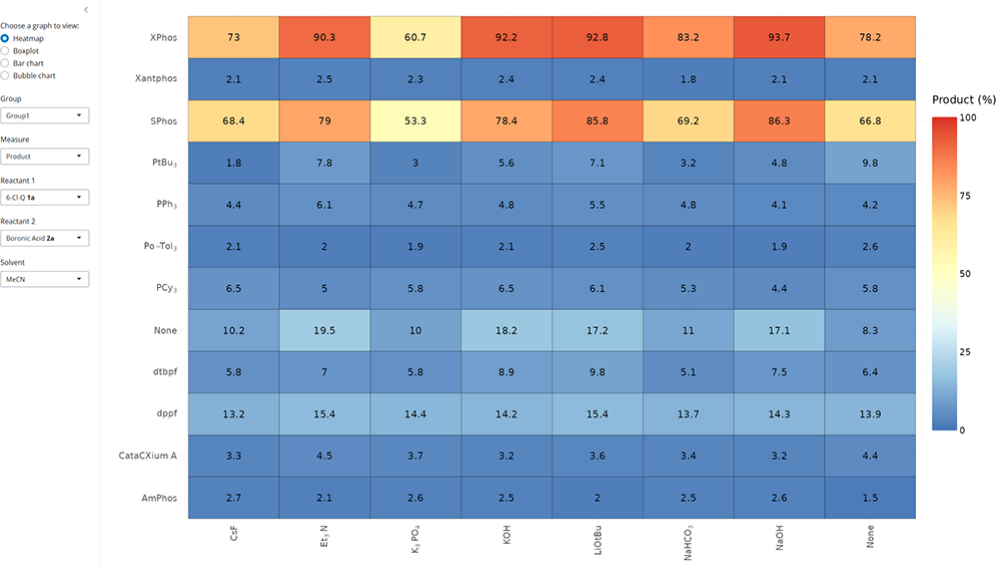
Screenshot of the Shiny app window, showing an example heatmap. The tab on the left displays the different parameters: four choices of graph, group choice, and the different measures associated with the graph selected. The left of the app contains the plotting window in which the graph selected will be displayed. A more detailed explanation of the app and its functions can be found within the Supporting Information.

### Principal Component Analysis (PCA)

PCA of the data further reveals interesting trends from the SMCC reaction data set. Figure 14 shows a PCA plot for the Group 1 data, showing the change in organohalide (6-Cl-Q **1a**, 6-Br-Q **1b,** 6-OTf-Q **1c,** and 6-I-Q **1d**), with the indazole boronic acid **2a** (plot A, Figure 14). Extended trends are evident, including the indazole boronic ester **2b** and indazole trifluoroborate salt **2c** derivatives (plot B, Figure 14). The maximum variance in the data is dominated by the balance of cross-coupling product **3** and protodeborylated side-product **2e**, highlighting the challenge of suppressing this in the reaction system under investigation. It is particularly evident that most side products are associated with indazole trifluoroborate salt **2c** (plot B, Figure 14). On the other hand, indazole boronic ester **2b** is generally a less reactive organoboron coupling partner in the reaction system.

**Figure 14:**
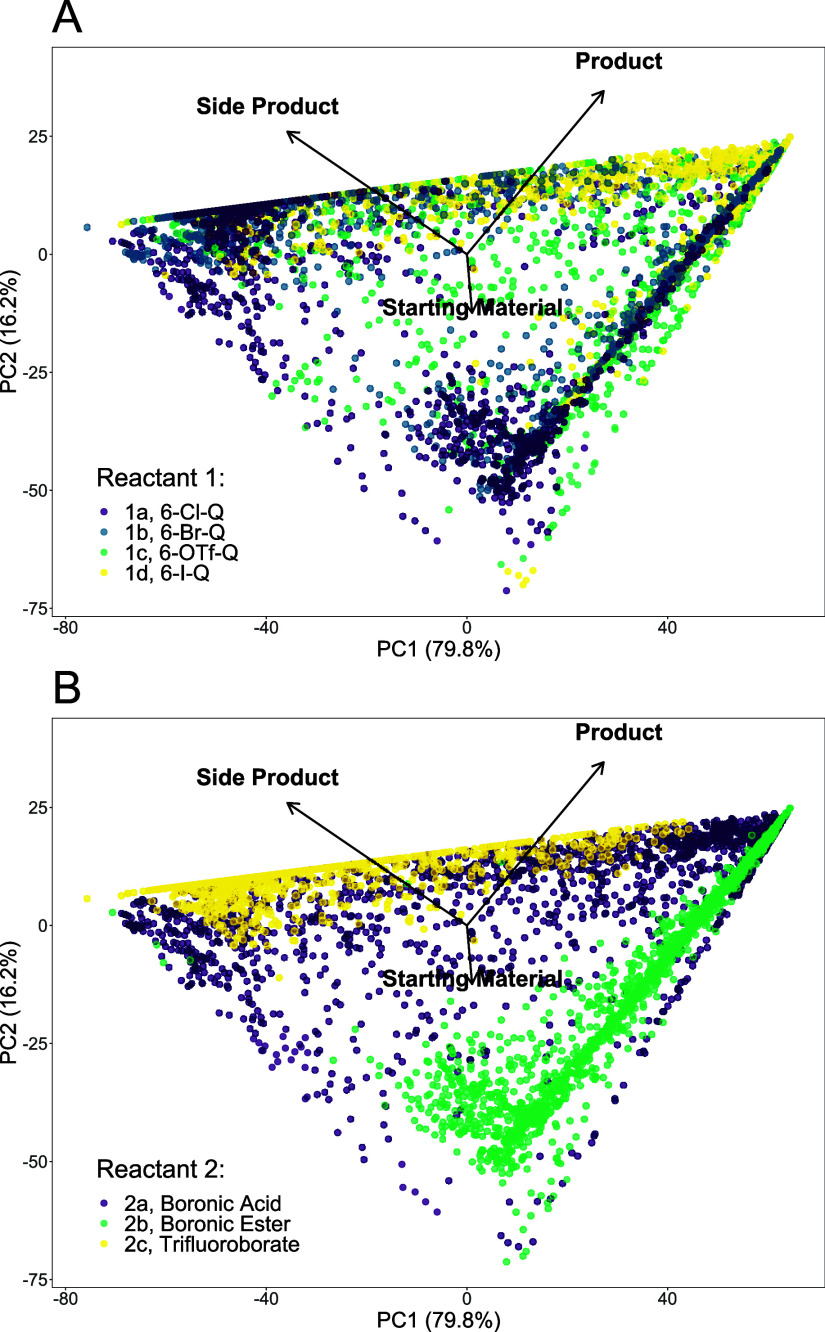
PCA of the reaction data set for Group 1. (A) Variance in the data set for changing organohalides **1a**–**1d**, employing indazole boronic acid **2a**. (B) Variance in the data set for changing organohalides **1a**–**1d**, employing indazole boron derivatives **2a**–**2c** (affording product **3** and/or side-product **2e**). For both figures, the variance explained by principal component 1 (PC1) is shown on the *x*-axis and the variance explained by principal component 2 (PC2) is shown on the *y*-axis. Figures 14A/B shows loadings arrows; the arrow directionality and length indicate how each variable contributes to the principal components.

## Conclusions

In this study, we have analyzed, arguably, perhaps the richest data set^7^ pertaining to a high-throughput screening campaign concerning Suzuki–Miyaura cross-couplings reported in the literature. The richness of these data cannot be overstated, with a diverse range of variables explored and key reaction outcomes reported. The data set is not without its limitations. For example, there are no technical replicates included within this data set (the purpose of the original study^7^ was the rapid identification of optimized conditions). Suspected coelution of the reaction components appears to have been one issue (by LCMS). With a data set this size, it is no surprise that over the course of some 5000+ reactions, there will always be some species that are unexpected and that do not resolve. That said, the instrumentation used to measure the data set is possibly more suited to prospecting reactions rather than identifying and understanding every side-product and byproduct present. However, its usefulness in this pursuit should not be underestimated.^7^

When considering the reactant choice in this data set, several trends emerged. For example, the quinoline halides **1a**–**1d** performed from best to worst in order of their reactivity: 6-Cl-Q **1a** worst; 6-Br-Q **1b**/6-OTf-Q **1c** performing similarly, and 6-I-Q **1d** the best in terms of the number of reactions with the highest amount of product **3** formed. When comparing the performance of indazoles **2a**–**2c**, there is a ‘worst’ clear choice for indazole trifluoroborate salt **2c** relative to **2a** and **2b**. As such, this meant that **2c** led to higher quantities of protodeborylation product **2e**.

By contrast, indazole boronic ester **2b** seemed to be the best indazole as it involved the fewest reactions that produced side-product **2e**. However, the mass balance of reactions containing indazole boronic ester **2b** was often lower than that of **2a** or **2c,** implying the presence of an unmeasured side product(s).

In the data set,^7^ there are two variables that have blanks included as options. One would expect the blank option to be the worst choice, as seen when examining the effect of the base chosen; however, the same cannot be said when examining the effects of the ligand choice on the data set. It was surprising to see that Xantphos was consistently the worst choice of ligand, being involved in fewer high-product-yielding reactions than no ligand at all. In fact, the bottom three ligands (not including blank) were Xantphos, dppf, and dtbpf, which are the only bidentate ligands included in this study. Conversely, when examining the results from the perspective of reaction conditions that lead to a higher quantity of side-product, the three bidentate ligands (Xantphos, dppf, and dtbpf) were involved, particularly when employing boronic acid **2a**.

There were two main perspectives considered when determining which ligand performed the best: considering high-yielding reactions, PPh_3_ is the best (*a surprising outcome for such a simple phosphine ligand*), with the caveat that PPh_3_ is a poor ligand for coupling 6-Cl-Q **1a**. Alternatively, when we considered utility across the substrate scope, it was determined that XPhos and SPhos were the best, despite their high quantities of reactions in the 35–65% range.

Throughout the detailed analysis undertaken as part of this study, one of the goals in mind was to identify trends in the reactions that produce cross-coupled product **3** in good to high yields. As analysis progressed and trends unfolded, there seemed to be no concrete set of optimal conditions, with even the worst-performing ligand, Xantphos, present in a reaction that yielded over 85% product. Moreover, indazole trifluoroborate **2c** was present in several reactions that yielded over 80% product despite performing worse than its related organoboron counterparts. It is, nevertheless, important to state that there is *no one-size-fits-all set of reaction conditions*. Rather, if one fixed a variable and optimized around it, then one would reach an optimum, even if it were a local one.

The key takeaway message of this exploration of data highlights the effectiveness of a high-throughput experimental screening campaign when corroborated with data analysis tools. The original data analyzed was collected at a rate of 1500 reactions per day.^7^ Through the use of data visualization tools, we have uncovered key details concerning the reaction variables that influence product and side-product yields. It was also possible to reveal missing products in the HTE data set. Such knowledge would have otherwise taken considerable time and effort to acquire via traditional approaches.

With the approach developed within this study, we hope that others will be encouraged to delve further into open reaction data sets produced by HTE campaigns.

## Supplementary Material



## Data Availability

Our shiny app can be found at https://shiny.york.ac.uk/UOY-SMCC1/ (hosting by the University of York).

## Notes

Synthetic chemistry details (experimental, including NMR spectral data and XRD data), description of Shiny app, and further analysis (PDF)

## Abbreviations

SMCC: Suzuki–Miyaura cross-coupling
HTE: high-throughput experimentation
