# Supplementary material for: Extracting Mechanistic Information from an Open Data Set for a Pharma-Relevant Suzuki–Miyaura Cross-Coupling Reaction
Source: Org Process Res Dev. 2026 Mar 6;30(3):568–77. doi: 10.1021/acs.oprd.5c00298 (PMC13010367; doi:10.1021/acs.oprd.5c00298)
Supplement: Supplementary file 1 [file op5c00298_si_001.pdf]

Extracting mechanistic information from an open dataset for a pharma-relevant Suzuki-Miyaura cross-coupling reaction

Barnabas A. Franklin,[a] Niall W. B. Donaldson,[a] James D. Firth,[a] Christopher S. Horbaczewskyj,[a] Aaron Aspell,[a] Theo Tanner,[a] Adrian C. Whitwood,[a] Julie Wilson,[b] Jessica K. Hargreaves[b,*] and Ian J. S. Fairlamb[a,*]

Author Addresses

[a] *Department of Chemistry, University of York, Heslington, York, North Yorkshire, YO10 5DD, United Kingdom. Email: ian.fairlamb@york.ac.uk*

[b] *Department of Mathematics, University of York, Heslington, York, North Yorkshire, YO105DD, United Kingdom. Email: jessica.hargreaves@york.ac.uk*

Electronic Supporting Information

Contents

Section 1: Experimental Details 3

1.1 General experimental details 3

1.2 Synthesis of reference materials for SMCC reactions 3

1.2.1 Synthesis of 4-bromo-5-methyl-1-(tetrahydro-2*H*-pyran-2-yl)-1*H*-indazole 2d 3

1.2.2 Synthesis of 6-(5-methyl-1-(tetrahydro-2H-pyran-2-yl) -1*H*-indazol-4-yl)quinoline 3 4

1.2.5 Synthesis of 5-methyl-1-(tetrahydro-2*H*-pyran-2-yl)-1H-indazol-4-yl boronic acid 2a 5

1.2.6 Synthesis of 5,5'-dimethyl-1,1'-bis(tetrahydro-2*H*-pyran-2-yl)-1*H*,1'*H*-4,4'-biindazole 5 6

1.2.7 Synthesis of 6,6'-biquinoline 4 6

1.2.8 Synthesis of 6-(5-methyl-1*H*-indazol-4-yl)quinoline S1 7

1.3 Representative NMR spectral data 8

1.4 Single crystal X-ray diffraction data 15

Section 2: Data analysis and generation of figures 20

2.1 Development of the Shiny App 20

2.1.1 User Interface 20

2.1.2 Heatmap generation 21

2.1.3 Boxplot generation 22

2.1.4 Bar chart generation 23

2.1.5 Bubble chart generation 24

2.2 Principal component analysis 25

2.3 Group 2 data analysis 25

Section 3: References 32

Section 1: Experimental Details

1.1 General experimental details

Reagents, where not synthesised, were purchased from the chemical suppliers Fluorochem, Sigma Aldrich, TCI, Arcos, and Alfa Aesar and used without further purification. Solvents were dried where required using the Puresolve PS-400-3-D solvent purification system from stock purchased from Fisher. Degassing where required was performed by sparging the solvent with N_2.

Purifications performed by manual flash column chromatography used Fisher solvents directly from the container; Sigma-Aldrich silica gel 60 was used. Thin-layer chromatography (TLC) plates used were Merck 5554 aluminium backed silica. Spots were visualised with short and long wave U.V. (254 and 365 nm respectively). A Teledyne Combiflash Rf using standard Redisep® Rf 4 and 12 g disposable silica columns was employed for automated column chromatography.

All NMR spectra were recorded on either a Jeol ECS400 or Bruker AVIII300NB, unless otherwise noted. Chemical shifts are reported in ppm calibrated to residual non-deuterated solvent shifts. Proton (^1H) spectra were recorded at 300 or 400 MHz as indicated, and proton decoupled carbon ($^{13}C\{^1H\}$) spectra were recorded at 75 or 101 MHz. Multiplicities are denoted as singlet (s), doublet (d), triplet (t), quartet (q), doublet of doublets (dd), doublet of triplets (dt) and multiplet (m). Coupling constants J are reported in Hz. All spectra were processed using MestReNova version 15.1.0.

Electrospray ionisation (ESI) mass spectrometry was performed using a Bruker Daltronics micrOTOF spectrometer. Mass to charge ratios (m/z) are reported in Daltons to 4 d.p.

Thin-film infrared spectra were recorded on a Perkin Elmer UATR Two FT-IR spectrometer. Absorption maxima (ν_{max}) are reported in wavenumbers (cm^{-1}) to the nearest whole number.

Diffraction data were collected at 110 K using an Oxford Diffraction SuperNova diffractometer with Cu-Kα radiation (λ = 1.54184 Å) using an EOS CCD camera. The crystal was cooled with an Oxford Instruments Cryojet. Diffractometer control, data collection, initial unit cell determination, frame integration and unit-cell refinement were carried out with “Crysalis”. Face-indexed absorption corrections were applied using spherical harmonics, implemented in SCALE3 ABSPACK scaling algorithm. OLEX2 was used for overall structure solution and refinement. Within OLEX2, the algorithm used for structure solution was “ShelXT dual-space”.[1] Refinement was carried out by full- matrix least-squares used the SHELXL-97[2] algorithm within OLEX2.[3] All non-hydrogen atoms were refined anisotropically. Crystalmaker® software was used to visualise the structures as well as generating the figures presented herein.

1.2 Synthesis of reference materials for SMCC reactions

1.2.1 Synthesis of 4-bromo-5-methyl-1-(tetrahydro-2*H*-pyran-2-yl)-1*H*-indazole 2d

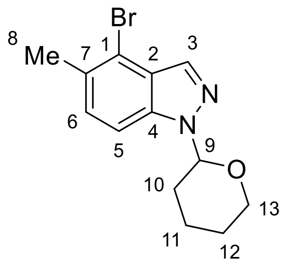

4-Bromo-5-methyl-1*H*-indazole (3.92 g, 18.5 mmol, 1.00 equiv.), pyridinium *p*-toluenesulfonate (550 mg, 2.19 mmol, 11 mol%), were dissolved in CH_2Cl_2 (60 mL) and sparged with N_2. 3,4-Dihydropyran (5.0 mL, 54.8 mmol, 2.96 equiv.) was added via syringe and the reaction mixture heated with stirring (35 °C) for 22 hours. The dark brown solution and was diluted with CH_2Cl_2 (20 mL). The organic layer was washed with saturated aqueous $NaHCO_3$ (3 × 20 mL) and dried over $MgSO_4$, filtered then concentrated *in vacuo* to give crude product **2d** as a brown solid. The product was purified by flash column chromatography

(SiO_2, 750 mL 9:1 *v*/*v* petroleum ether: EtOAc), to afford, following evaporation of the pure fractions, a colourless solid **2d** (4.85 g, 89%).

^{1}H NMR (400 MHz, chloroform-*d*) δ 7.97 (*H*3, 1H, s), 7.43 (*H*6, 1H, d, J = 8.5 Hz), 7.23 (*H*5, 1H, d, J = 8.5 Hz), 5.67 (THP-*H*9, 1H, dd, J = 9.2, 2.8 Hz), 3.97–4.02 (THP-*H*13 *ax*., 1H, m), 3.69-3.75 (THP-*H*13 *eq*., 1H, m), 2.50–2.57 (THP, 1H, m), 2.48 (*H*8, 3H, s), 2.10–2.17 (THP, 1H, m), 2.02–2.08 (THP, 1H, m), 1.62–1.79 (THP protons, 3H, m).

^{13}C{^{1}H} NMR (101 MHz, chloroform-*d*) δ 138.86 (C1), 133.95 (C3), 130.18 (C2/C7), 129.63 (C5/C6), 126.61 (C2/C7), 115.54 (C4), 109.21 (C5/C6), 85.75 (C9), 67.47 (C13), 29.47 (C10/C11/C12), 25.19 (C10/C11/C12), 22.54 (C10/C11/C12), 21.61 (C8).

IR (ATR) 2960 (CH), 2930 (CH), 2850 (CH), 1421 (CC), 1172, 1078, 797 (CH def.) cm^{-1}.

MS (ESI) 317.0267 [M+Na] (^{79}Br, calc. 317.0265, 0.63 ppm error).

MP 92 °C.

1.2.2 Synthesis of 6-(5-methyl-1-(tetrahydro-2H-pyran-2-yl) -1*H*-indazol-4-yl)quinoline 3

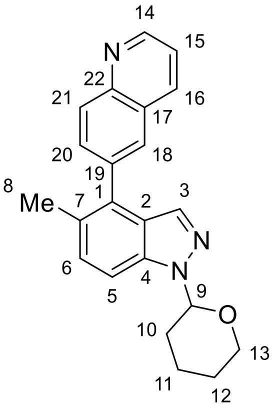

To a vial was added bromoindazole **2d** (51.3 mg, 0.17 mmol, 1.00 equiv.), 6-quinolineboronic acid (31.3 mg, 0.18 mmol, 1.05 equiv.), $Pd(OAc)_2$ (2.7 mg, 0.0120 mmol, 7 mol%), PPh_3 (6.0 mg, 0.023 mmol, 13 mol%), $NaHCO_3$ (37.6 mg, 0.448 mmol, 2.87 equiv.). The vial was purged with N_2, then with magnetic stirring, THF (0.9 mL) and H_2O (0.1 mL) were added. The reaction mixture was heated to 85 °C and stirred for 18 hours. The solution was diluted with EtOAc (5 mL) and washed with aqueous $NaHCO_3$ (3 × 5 mL) and the organic layer was dried over $MgSO_4$, filtered and concentrated *in vacuo* to give the crude product as a brown oil. The title compound was purified via flash column chromatography (SiO_2, 500 mL 1:1 EtOAc: Petroleum ether, 250 mL 3:1 *v*/*v* EtOAc: petroleum ether, EtOAc), 150 mL), to afford, following evaporation of the pure fractions, product **3** as a solid (50.1 mg, 84%). Single crystals were for XRD analysis were grown from Et_2O by slow evaporation.

^{1}H NMR (600 MHz, MeOD) δ 9.06 (*quinonyl*, 1H, dd, J = 4.3, 1.7 Hz), 8.60 (*H5/6*, 1H, dt, J = 8.4, 1.3 Hz), 8.33 (*H5/6*, 1H, d, J = 8.7 Hz), 8.14 (*H*3, 1H, d, J = 1.9 Hz), 7.98 (*quinonyl*, 1H, dd, J = 8.7, 2.0 Hz), 7.81–7.78 (*quinonyl*, 2H, m), 7.76 (*quinonyl,* 1H, dd, J = 8.3, 4.3 Hz), 7.59 (*quinonyl*, 1H, d, J = 8.6 Hz), 5.98 (*H*9, 1H, dd, J = 9.9, 2.6 Hz), 4.20–4.16 (THP proton, 1H, m), 3.88–3.80 (THP proton, 1H, m), 3.98 (td, *J* = 11.3, 2.9 Hz, 1H), 2.70–2.60 (THP proton, 1H, m), 2.28 (H8, 3H, s), 2.21-2.15 (THP proton, 1H, m), 2.00 (THP proton, 1H, dd, J = 12.9, 3.5 Hz), 2.00 (THP proton, 1H, dtt, J = 13.3, 11.9, 4.1 Hz), 1.93–1.78 (THP protons, 2H, m).

^{13}C{^{1}H} NMR (151 MHz, MeOD) δ 151.56, 148.02, 139.91, 138.86, 138.57, 134.01, 133.62, 133.14, 131.34, 129.92, 129.79, 129.37, 129.27, 126.42, 123.05, 110.80, 86.55, 68.74, 30.69, 26.35, 23.78, 19.39.

MS (ESI) 344.1761 [M+H] (calc. 344.1763, error 0.58 ppm).

IR (ATR) 2926 (CH), 2856 (CH), 1493 (CC), 1440 (CC), 1081 (CO), 1042 (CC), 840 (CC), 798 (CC) cm^{-1}.

MP 136-138 °C

The molecular structure was confirmed by single crystal X-Ray crystallography.

1.2.3 Synthesis of 5-methyl-1-(tetrahydro-2*H*-pyran-2-yl)-1*H*-indazole 2e

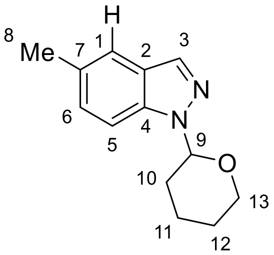

To a flask, indazole **2d** (152 mg, 0.51 mmol, 1.00 eq) and dry THF (5 mL) was added. The flask was purged with N_2 and cooled to –78 °C. With stirring, *n*-BuLi (2.5 M in hexanes, 5.0 mL, 12.5 mmol, 24.5 eq.) was added dropwise and the resulting solution was stirred at – 78 °C for 1 hour. The reaction was quenched with aqueous NH_4Cl (5 mL) and allowed to warm to room temperature. The solution was diluted with EtOAc (5 mL) and washed with aqueous $NaHCO_3$ (3 × 5 mL) and the organic layer was dried over $MgSO_4$, filtered and concentrated *in vacuo* to give brown oil crude product. Product was purified via flash column chromatography (SiO_2, 400 mL 1:4 EtOAc: Petroleum ether) to afford **2e** as a yellow oil (41.8 mg, 42%)

^{1}H NMR (400 MHz, MeOD) δ 7.90 (H3, 1 H, s), 7.50 (H5/6, 1H, d, J = 8.6 Hz), 7.45 (H1, 1 H, s), 7.22 (H5/6, 1H, dd, J = 8.7, 1.7 Hz), 5.68 (H9, 1H, dd, J = 10.0, 2.6 Hz), 4.00–3.90 (H13, 1H, m), 3.73 (H13, 1H, td, J = 11.2, 2.8 Hz), 2.52 – 2.39 (THP, 1H, m), 2.40 (H8, 3H, s), 2.10 – 2.00 (THP, 1H, m), 1.96 – 1.88 (THP, 1H, m), 1.84 – 1.44 (THP, 3H, m).

^{13}C{^{1}H} NMR (101 MHz, MeOD) δ 139.60 (*C*1), 134.39 (*C*3), 132.05 (*C*2/7), 129.91 (*C*5/6), 126.17 (*C*2/7), 120.95 (*C*4), 110.89 (*C*5/6), 86.33 (*C*9), 68.62 (*C*13), 30.60 (*C*10/11/12), 26.26 (*C*10/11/12), 23.76 (*C*10/11/12), 21.29.

IR (ATR) 2939 (CH), 2859 (CH), 1510 (sh)(CC), 1439 (CC), 1210 (CH), 1082 (CO), 1043 (CH), 936 (CH) cm^{-1}

MS (ESI) 239.1154 [M+Na] (calc. 239.1160, error 2.51 ppm).

1.2.5 Synthesis of 5-methyl-1-(tetrahydro-2*H*-pyran-2-yl)-1H-indazol-4-yl boronic acid 2a

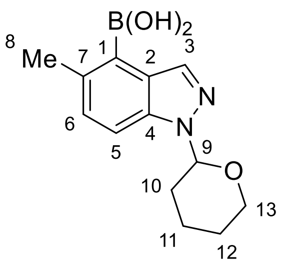

To a round bottomed flask under a N_2 atmosphere was added bromoindazole **2d** (732 mg, 3.47 mmol, 1.00 equiv.) and triisopropyl borate (1.18 mL, 5.10 mmol, 1.46 equiv.). Dry THF (7 mL) was added via cannula on a Schlenk line. The flask was cooled to –78 °C. With magnetic stirring, *n*-BuLi (2.5 M in hexanes, 1.9 mL, 4.75 mmol, 1.37 equiv.) was added dropwise over the course of 30 minutes, with care that the surrounding bath did not warm to above –65 °C. The reaction was quenched slowly with aqueous NH_4Cl (7 mL) and allowed to warm to room temperature. The solution was diluted with MTBE (7 mL) and washed with water (3 × 5 mL) and the organic layer was dried over $MgSO_4$, filtered and concentrated *in vacuo* to give the crude product as a viscous orange oil. The product was purified using automated column chromatography (SiO_2, 10% to 90% *v*/*v* EtOAc/petroleum ether), to afford, following combination of the pure fractions, the title compound **2a** as an off-white solid (696 mg, 71%). Single crystals suitable for XRD analysis were crystalised from MeOH by slow evaporation.

^{1}H NMR (400 MHz, MeOD) δ 7.93 (*H*3, 1H, d, J = 9.1 Hz), 7.67 (*H*5/6, 1H, t, J = 9.3 Hz), 7.38 (*H*5/*H*6 1H, t, J = 9.3 Hz), 5.87 (*H*9, 1H, app. t, J = 9.3 Hz), 4.10 (*H*9, 1H, s), 3.90 (THP proton, 1H, d, J = 9.6 Hz), 2.65–2.45 (THP proton *and H8*, 4H, m), 2.25–2.12 (THP proton, 1H, m), 2.12–2.04 (THP proton, 1H, m),

2.00–1.68 (THP proton, 3H, m), 1.34 (THP proton, 1H, s), 1.21 (THP proton, 1H, d, J = 10.3 Hz), 0.99–0.83 (THP proton, 1H, m).

^{11}B NMR (128 MHz, MeOD) δ 29.21.

IR(ATR) 3416 (br)(OH), 2942 (CH), 2859 (CH), 1420 (CH), 1376 (CH), 1040 (CO), 998 (CH) cm^{-1}.

MS (ESI) 283.1228 [M+Na] (^{11}B, Calc. 283.1230, 0.71 ppm).

MP 112-118 °C.

The molecular structure was confirmed by single crystal X-Ray crystallography. No ^{13}C{^{1}H} was recorded for this compound.

1.2.6 Synthesis of 5,5'-dimethyl-1,1'-bis(tetrahydro-2*H*-pyran-2-yl)-1*H*,1'*H*-4,4'-biindazole 5

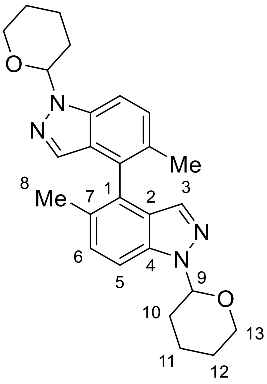

To a vial was added indazole boronic acid **2a** (150 mg, 0.577 mmol, 1.00 equiv.), bromoindazole **2d** (225 mg, 0.865 mmol, 1.50 equiv.), $Pd_2(dba)_3$ (17.3 mg, 0.0170 mmol, 3 mol%), SPhos (13.5 mg, 0.0330 mmol, 6 mol%), and K_3PO_4 (236 mg, 1.11 mmol, 1.92 equiv.). The vial was purged with N_2, and dry, degassed toluene (2 mL) was added. The reaction was heated to 100 °C and magnetically stirred for 20 hours to give a brown solution of a suspended precipitate. The solution was diluted with EtOAc (10 mL), then washed with aqueous $NaHCO_3$ (3 × 10 mL). The organic layer was dried over $MgSO_4$, filtered and concentrated *in vacuo*. The title compound **5** was isolated by automated column chromatography as colourless oil (SiO_2, 10% to 90% EtOAc in Petroleum ether) (231.6 mg, 62%). Three species seen by NMR spectroscopic analysis, likely due to diastereomers and conformational isomerism (atropisomers).

^{1}H NMR (400 MHz, chloroform-*d*) δ 7.54 (1H, dd, J = 8.6, 4.7 Hz), 7.44–7.16 (2H, m), 5.76–5.69 (1 H, m), 4.09 (1H, dd, J = 10.8, 3.8 Hz), 3.77 (1H, ddd, J = 11.2, 8.8, 2.6 Hz), 2.63–2.49 (5H, m), 2.11 (1H, t, J = 5.0 Hz), 1.84–1.66 (2H, m). Assignment of spectra not possible due to presence of overlapping peaks from multiple species (atropisomers and diastereomers).

^{13}C{^{1}H} NMR (101 MHz, chloroform-*d*) δ 138.32, 138.27, 133.69, 133.64, 133.57, 130.11, 129.79, 129.73, 128.88, 125.25, 125.20, 109.35, 109.21, 85.75, 85.62, 85.58, 67.97, 67.94, 67.84, 29.67, 29.65, 29.60, 25.30, 22.99, 22.91, 18.85, 18.83. Assignment of spectra not possible due to overlapping peaks from multiple species (atropisomers and diastereomers).

MS (ESI) 431.2457 [M+H] (calc. 431.2447, 2.32 ppm).

1.2.7 Synthesis of 6,6'-biquinoline 4

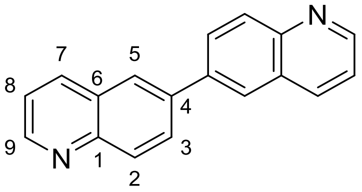

To a vial was added 6-quinolineboronic acid **1e** (196 mg, 1.14 mmol, 1.00 equiv.), $Pd(OAc)_2$ ($Pd_3(OAc)_6$ form, 14.1 mg, 0.0627 mmol, 6 mol%), PPh_3 (35.6 mg, 0.135 mmol, 12 mol%), $NaHCO_3$ (310 mg, 3.69

mmol, 3.24 equiv.). The vial was purged with N_2, then with stirring, THF (1.8 mL), and H_2O (0.2 mL) were added. 6-Bromoquinoline **1b** (0.15 mL, 1.16 mmol, 1.02 equiv.) was added and the reaction mixture heated for 18 hours at 80°C. The solution was diluted with EtOAc (5 mL) and washed with H_2O (3 × 5 mL). The organic layer was dried over $MgSO_4$, filtered and concentrated *in vacuo* to give the crude product as a brown oil. The title compound **4** was purified via flash column chromatography (SiO_2, 500 mL EtOAc), to afford, following combination of the pure fractions, a fine colourless crystalline powder (153 mg, 52%). A sample was crystalized from $CHCl_3$ by slow evaporation and a single crystal XRD structure determined.

^{1}H NMR (300 MHz, chloroform-*d*) δ 8.96 (1H, dd, J = 4.3, 1.7 Hz), 8.30 – 8.21 (2 H, m), 8.16 – 8.09 (2 H, m), 7.48 (1 H, dd, J = 8.3, 4.2 Hz).

^{13}C{^{1}H} NMR (101 MHz, chloroform-*d*) δ 150.85, 147.98, 138.55, 136.46, 130.35, 129.35, 128.65, 126.22, 121.84, 77.16.

IR (ATR) 3064 (CH), 3010 (CH), 1582 (CC), 1570 (CC), 1485 (CH), 1335 (CH), 1127 (CN), 871 (CH), 826 (CH), 793 (CH), 766 (CH), 620 (CH) cm^{-1}.

MS (ESI) 257.1076 [M+1] (calc. 257.1079, error 1.17 ppm).

MP 178-186 °C.

The molecular structure was confirmed by single crystal X-Ray crystallography.

1.2.8 Synthesis of 6-(5-methyl-1*H*-indazol-4-yl)quinoline S1

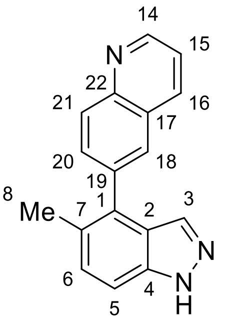

To a flask was added compound **2** (28.7 mg, 0.0835 mmol, 1.00 equiv.), CH_2Cl_2 (2 mL), and TFA (0.5 mL) was added. The reaction was stirred at room temperature for 18 hours. The yellow solution was neutralised with saturated aqueous $NaHCO_3$ dropwise, diluted with CH_2Cl_2 (5 mL) and washed with $NaHCO_3$ (3 × 5 mL). The organic layer was dried over $MgSO_4$, filtered and concentrated *in vacuo* to give the crude product as a yellow oil. The title compound was purified by flash column chromatography (SiO_2, 500 mL 20% *v*/*v* Petroleum ether in EtOAc), to give title compound **S1** as a colourless oil (13.4 mg, 61%).

^{1}H NMR (600 MHz, MeOD) δ 9.05 (dd, *J* = 4.3, 1.7 Hz, 1H), 8.58 (*quinonyl*, dt, *J* = 8.4, 1.2 Hz, 1H), 8.31 (*quinonyl*, d, *J* = 8.6 Hz, 1H), 8.13 (*quinonyl*, d, *J* = 1.9 Hz, 1H), 7.98 (*quinonyl*, dd, *J* = 8.6, 1.9 Hz, 1H), 7.78 *quinonyl*, (s, 1H), 7.74 (*quinonyl*, dd, *J* = 8.3, 4.3 Hz, 1H), 7.64 (*quinonyl*, d, *J* = 8.5 Hz, 1H), 7.53 (d, *J* = 8.6 Hz, 1H), 2.49 (8, s, 3H). *Selected peaks shown from spectra due to intractable mixture.*

^{13}C{^{1}H} NMR (151 MHz, MeOD) δ 151.48, 147.96, 140.61, 139.24, 138.56, 133.81, 133.21, 131.36, 129.92, 129.72, 129.19, 128.50, 125.01, 123.01, 110.69, 49.00, 19.45. *Selected peaks shown from spectra due to intractable mixture.*

IR (ATR) 3160 (CH), 3054 (CH), 2931 (CH), 2861 (CH), 2779 (CH), 1494 (CC), 1360, 1265, 1080, 1025, 950 (CH deformation), 842 (CH deformation), 799 (CH deformation) cm^{-1}.

MS (ESI) 260.1182 [M+1] (calc. 260.1188, 2.31 ppm).

1.3 Representative NMR spectral data

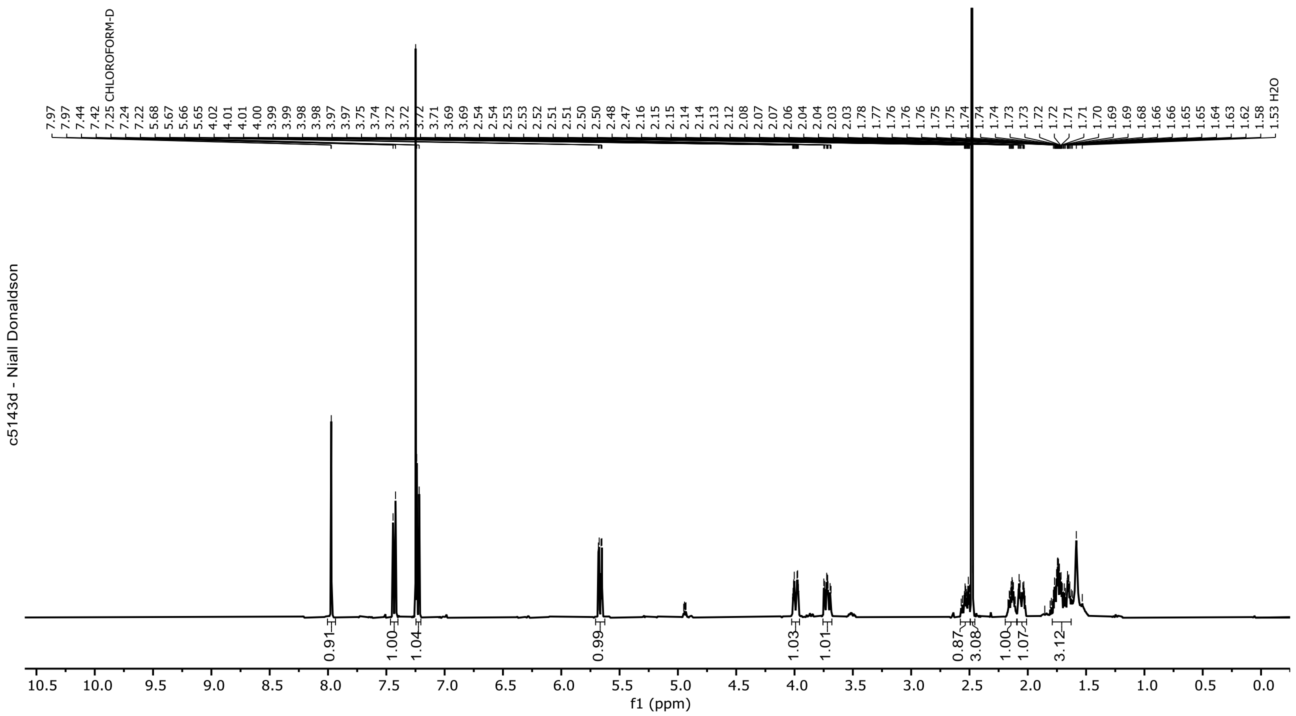

Figure S1 - Representative ^{1}H NMR spectrum of compound **2d** (in $CDCl_3$).

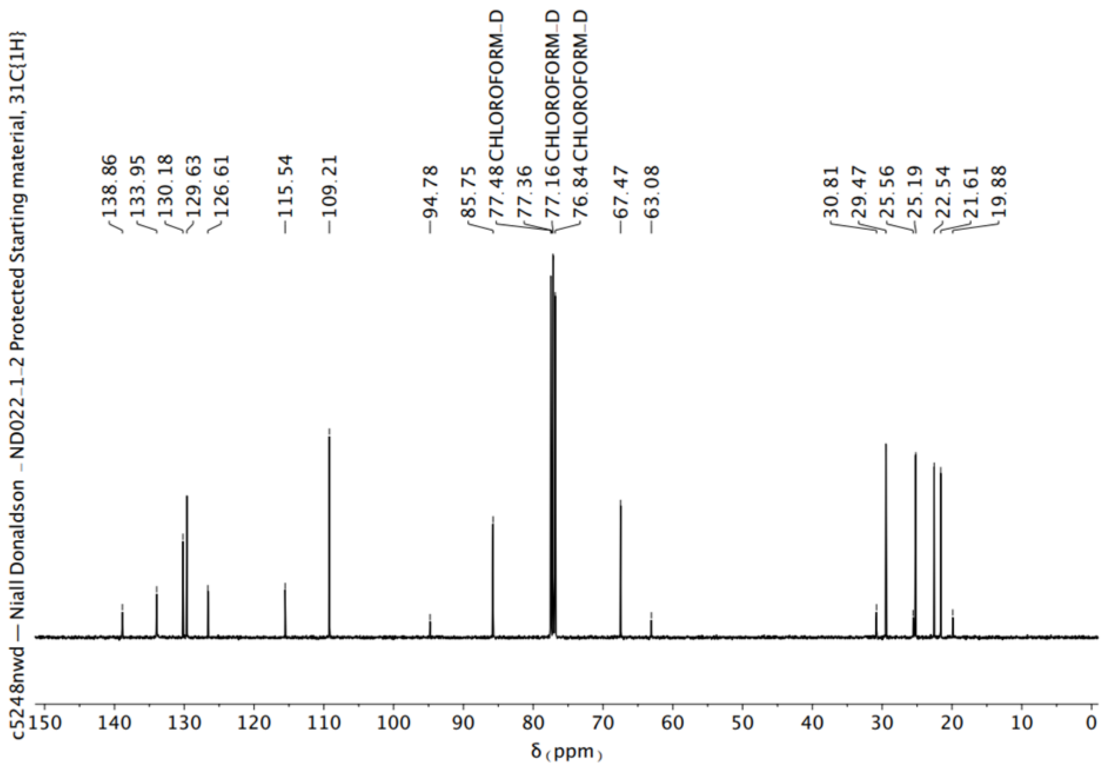

Figure S2 - Representative $^{13}C\{^{1}H\}$ NMR spectrum of compound **2d** (in $CDCl_3$).

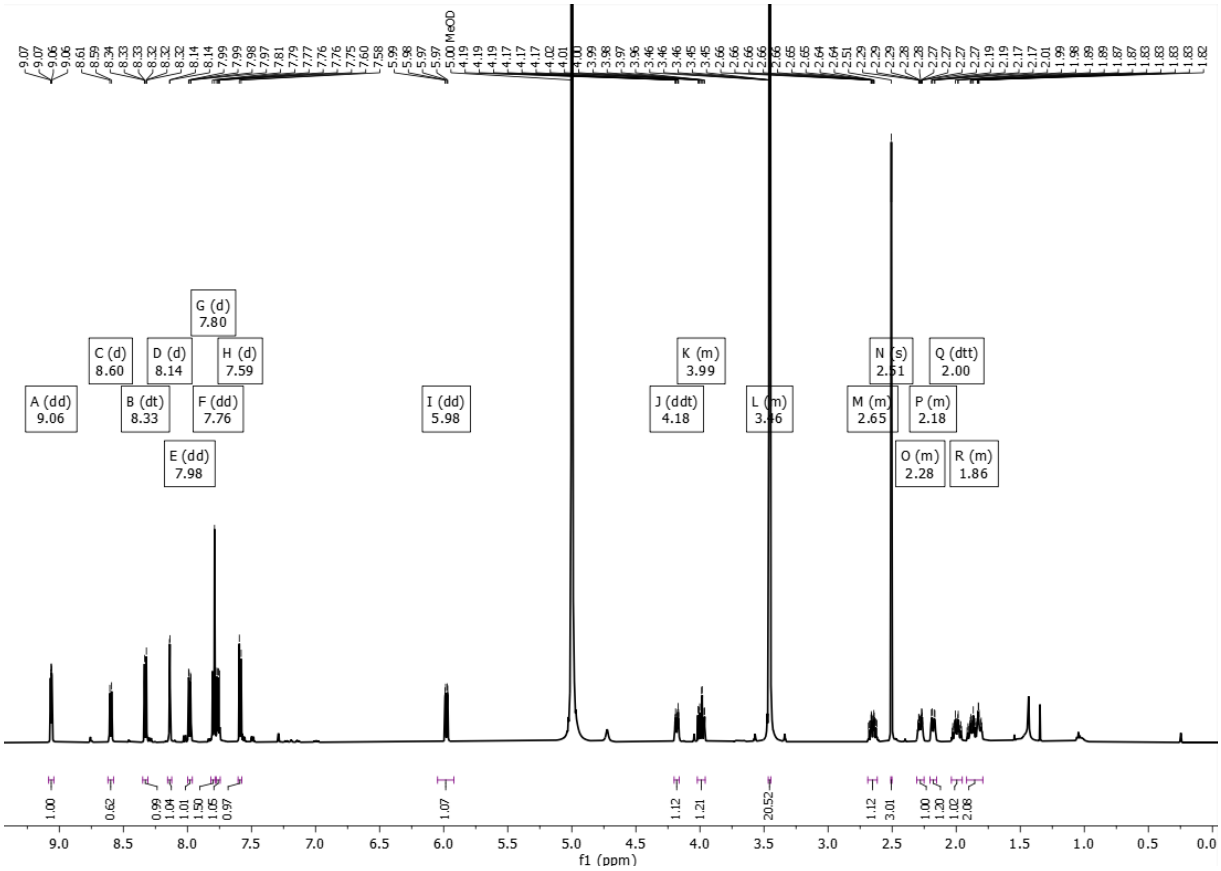

Figure S3 - Representative ^{1}H NMR spectrum of compound **3** (in Methanol-d_4).

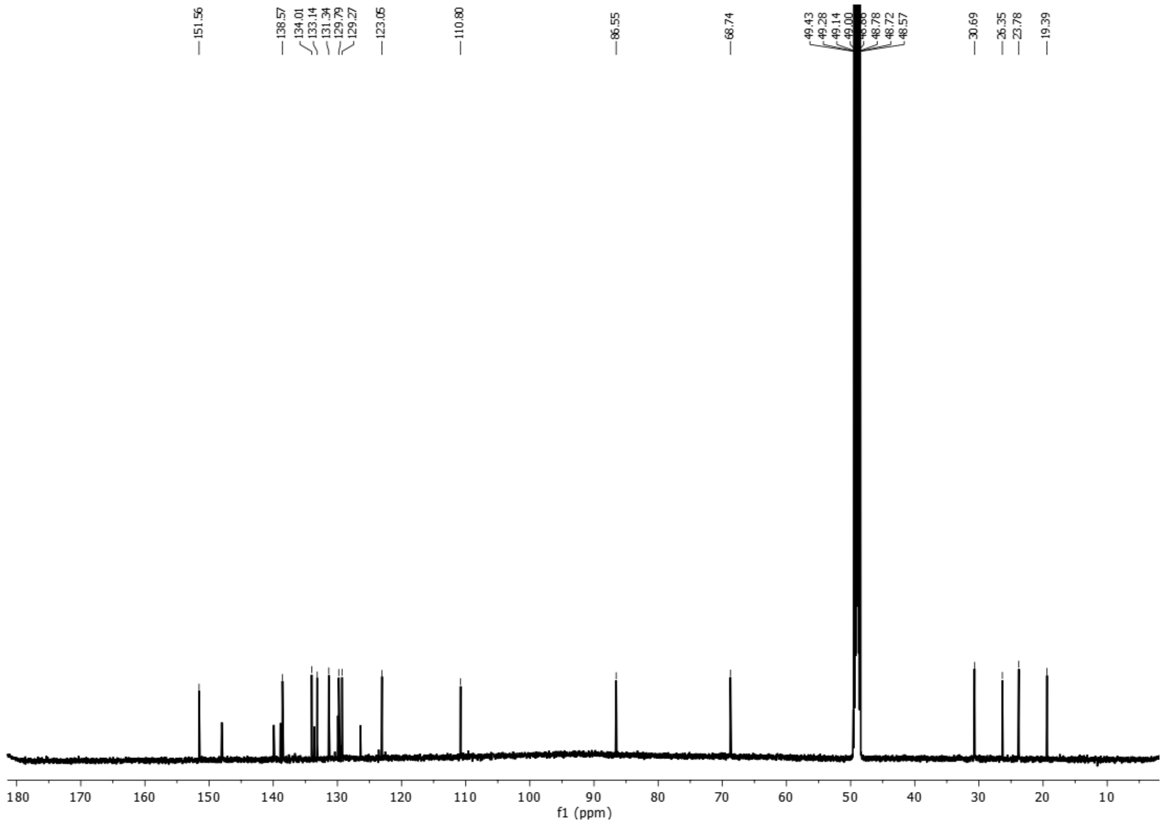

Figure S4 - Representative $^{13}C\{^{1}H\}$ NMR spectrum of compound **3** (in Methanol-d_4).

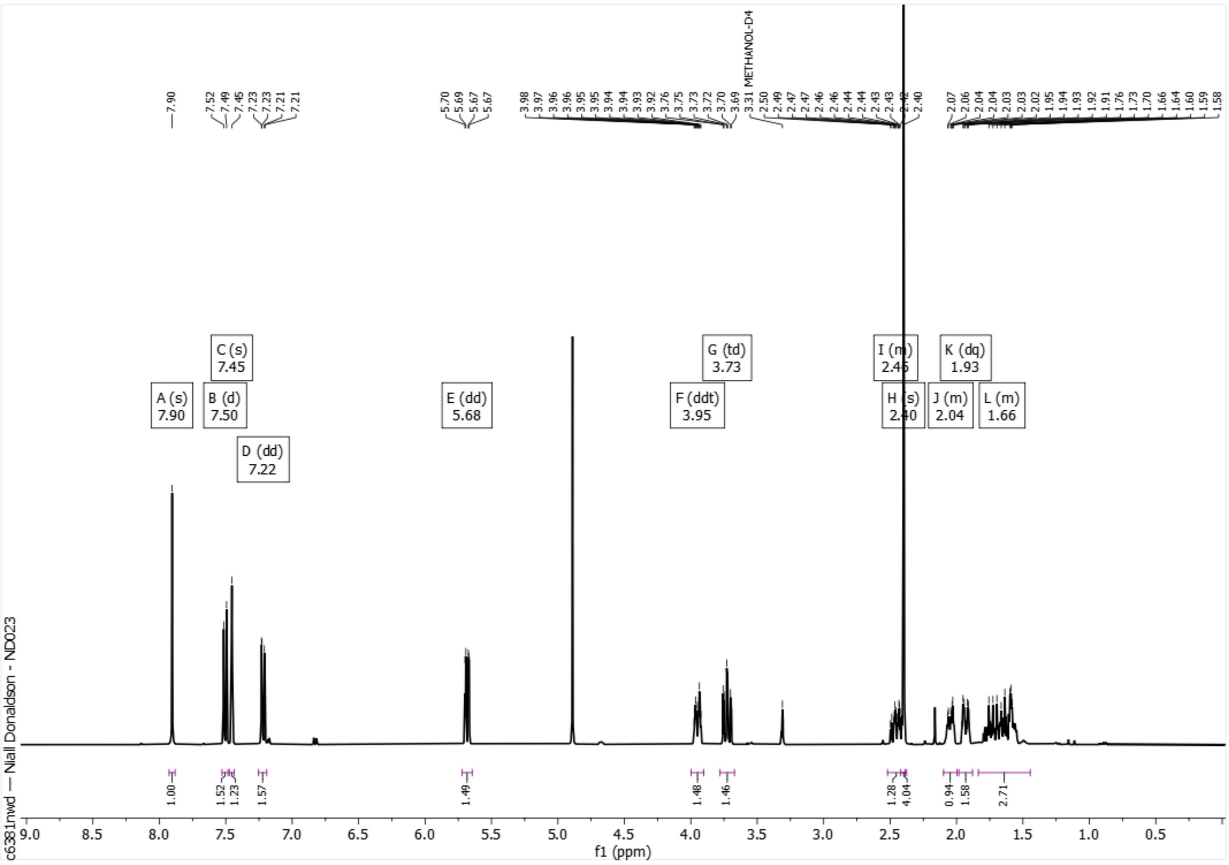

Figure S5 - Representative ^{1}H NMR spectrum of compound **2e**(in Methanol-d_4).

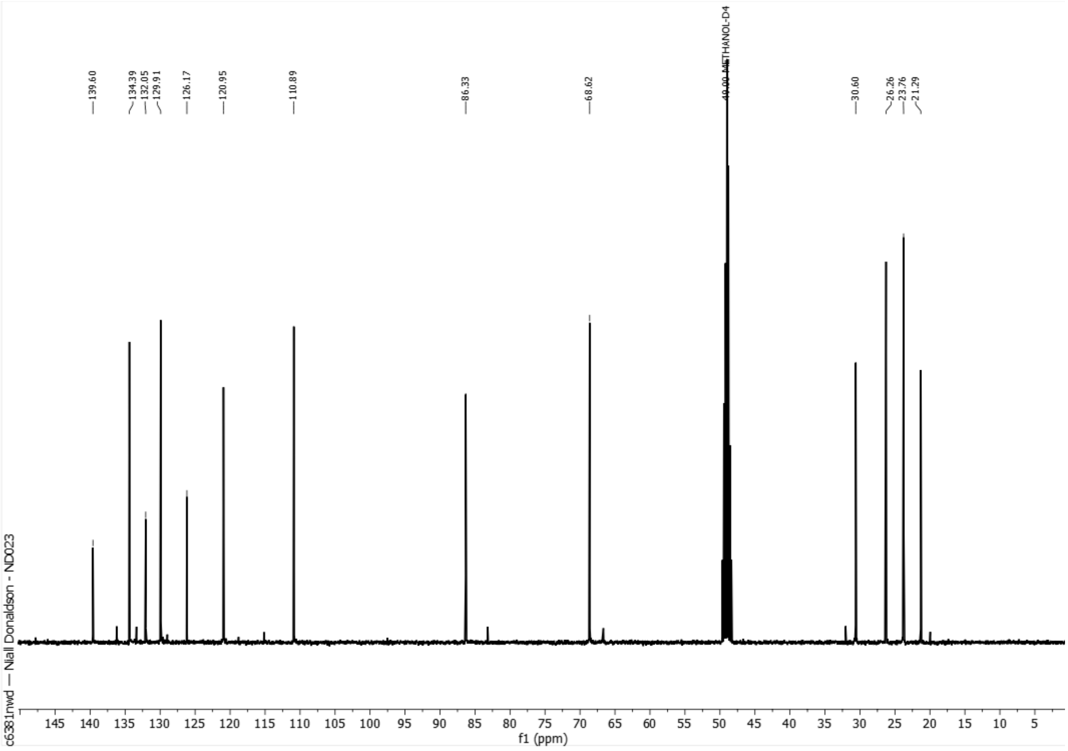

Figure S6 - Representative ^{13}C{^{1}H} NMR spectrum of compound **2e** (in Methanol-d_4).

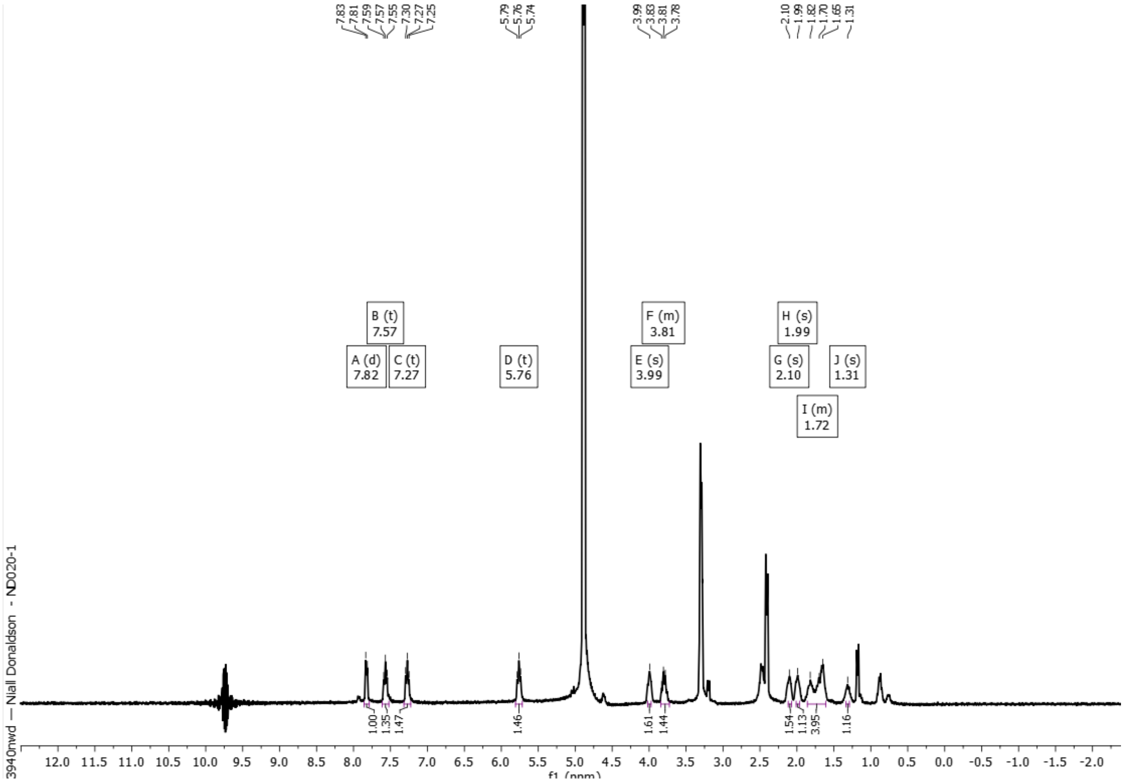

Figure S7 - Representative ^{1}H NMR spectrum of impure compound **2a** (in Methanol-d_4). Presence of artefact at 9.70 ppm is due to technical fault of the spectrometer.

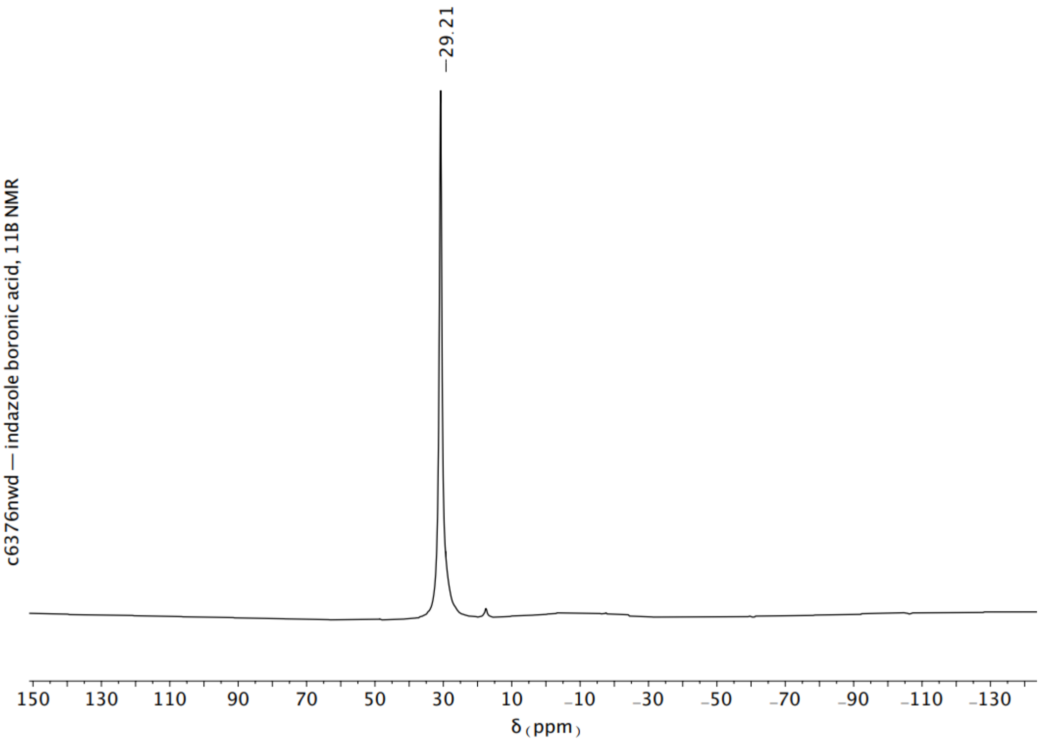

Figure S8 - Representative ^{11}B{^{1}H} NMR spectrum of compound **2a** (in Methanol-d_4).

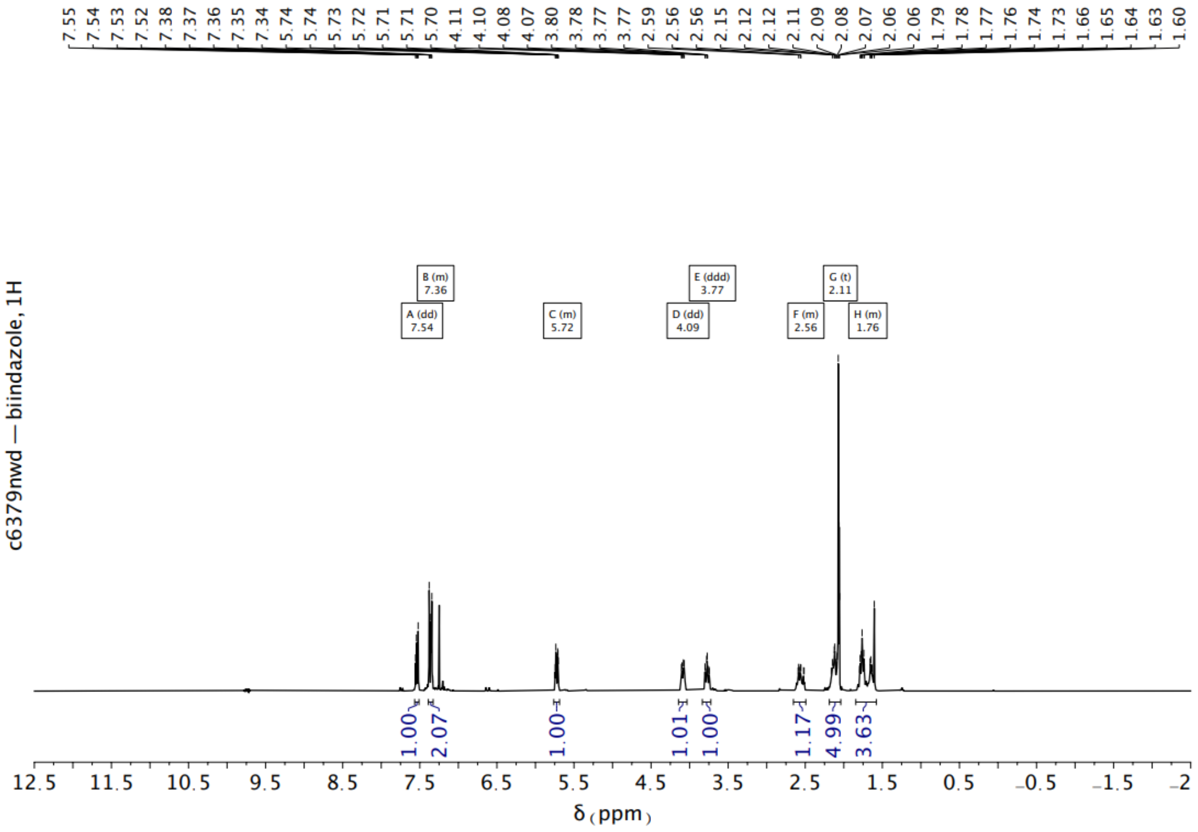

Figure S9 - ^{1}H NMR spectrum of compound **5** (in $CDCl_3$).

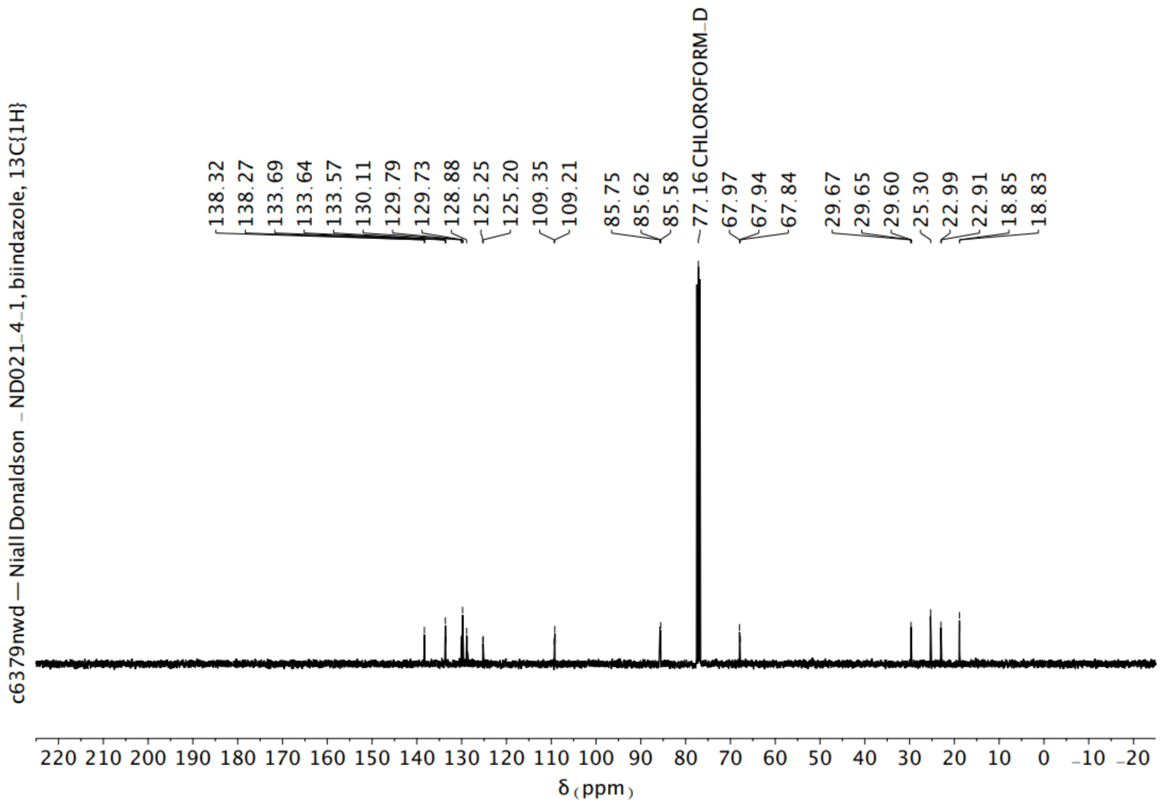

Figure S10 - Representative $^{13}C\{^{1}H\}$ NMR spectrum of compound **5** (in $CDCl_3$).

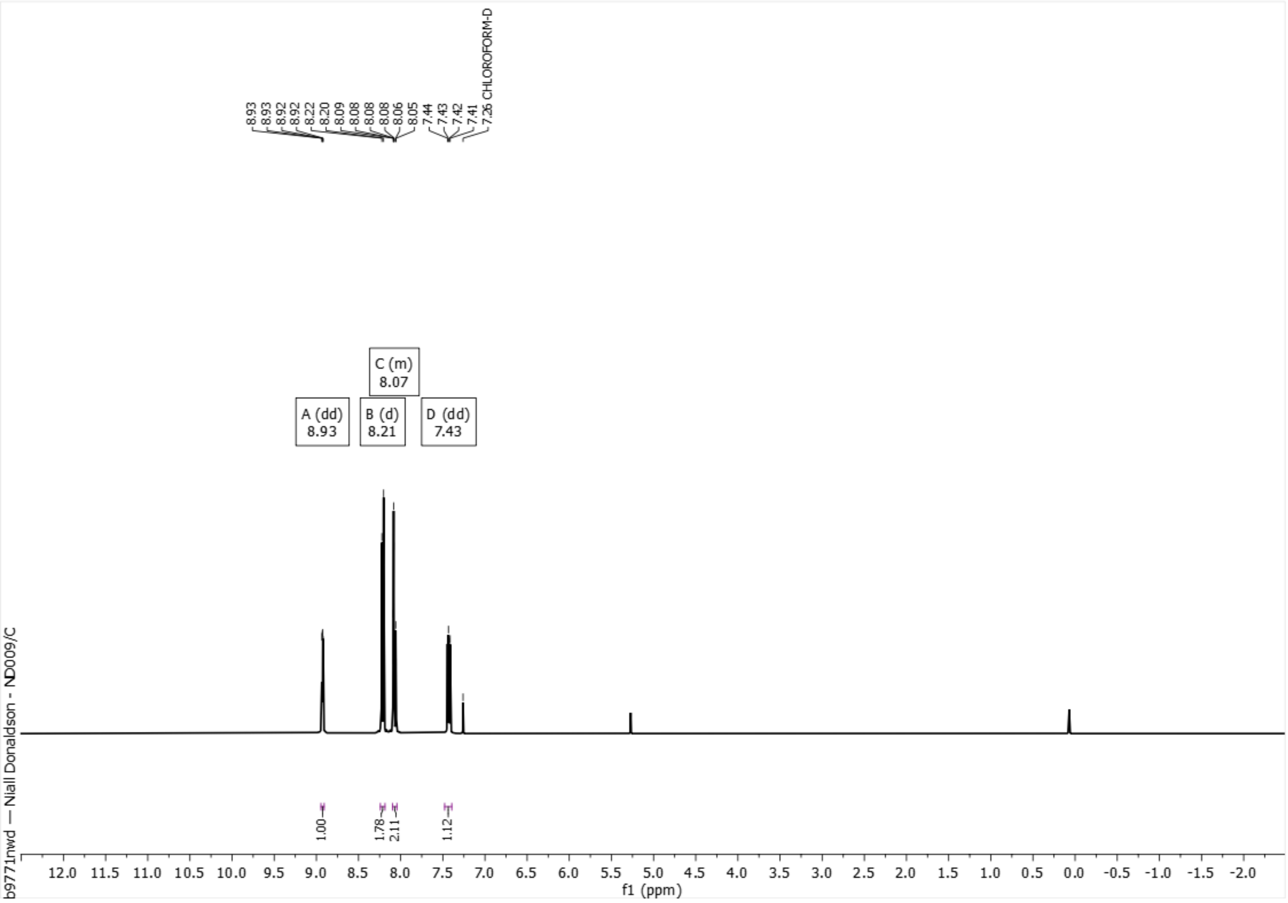

Figure S11 - ^{1}H NMR spectrum of compound **4** (in $CDCl_3$).

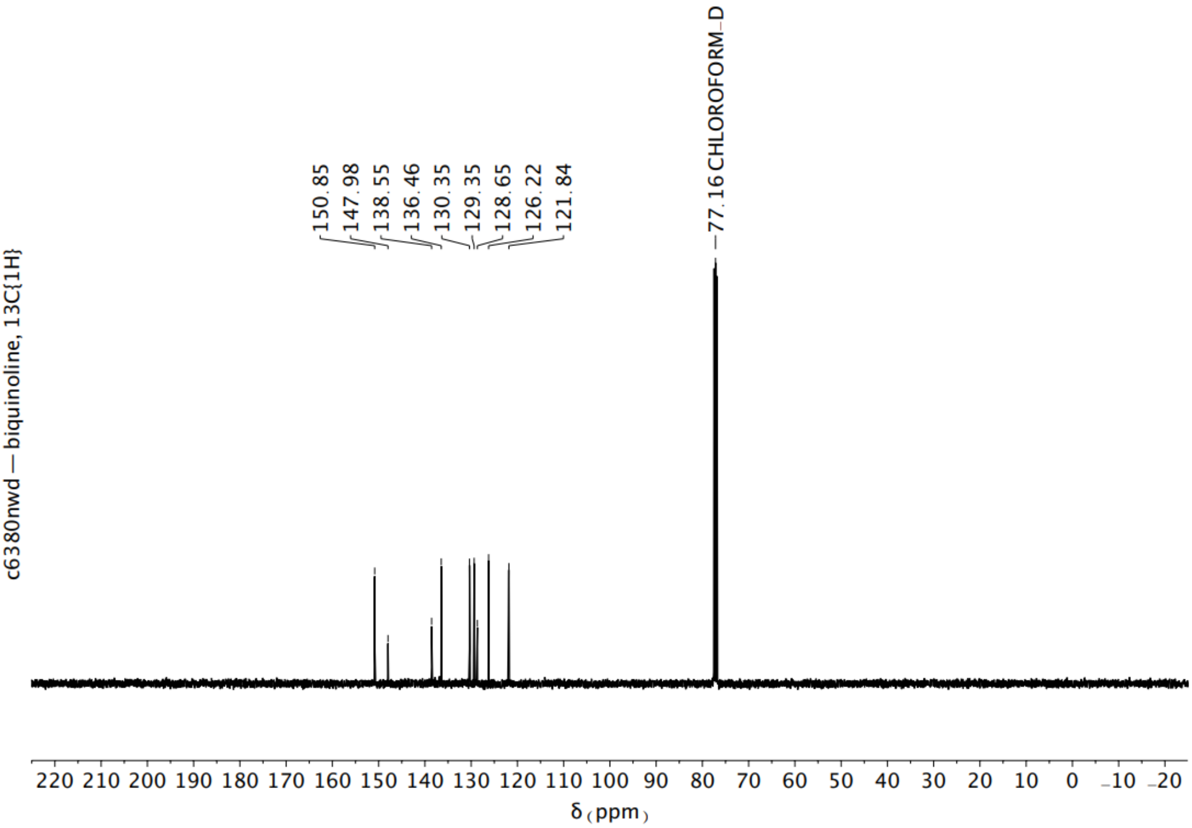

Figure S12 - Representative $^{13}C\{^{1}H\}$ NMR spectrum of compound **4** (in $CDCl_3$).

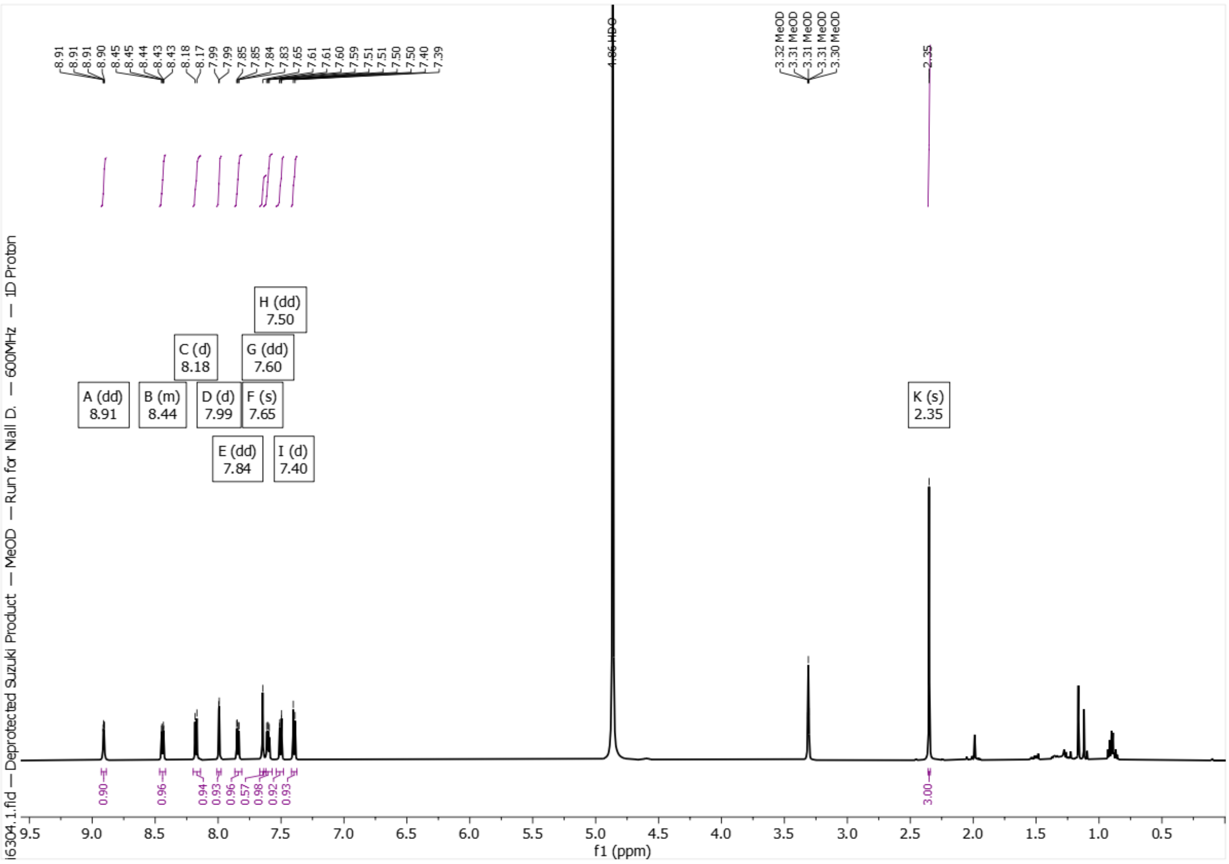

Figure S13 - Representative ^{1}H NMR spectrum of compound **S1** (in Methanol-d_4).

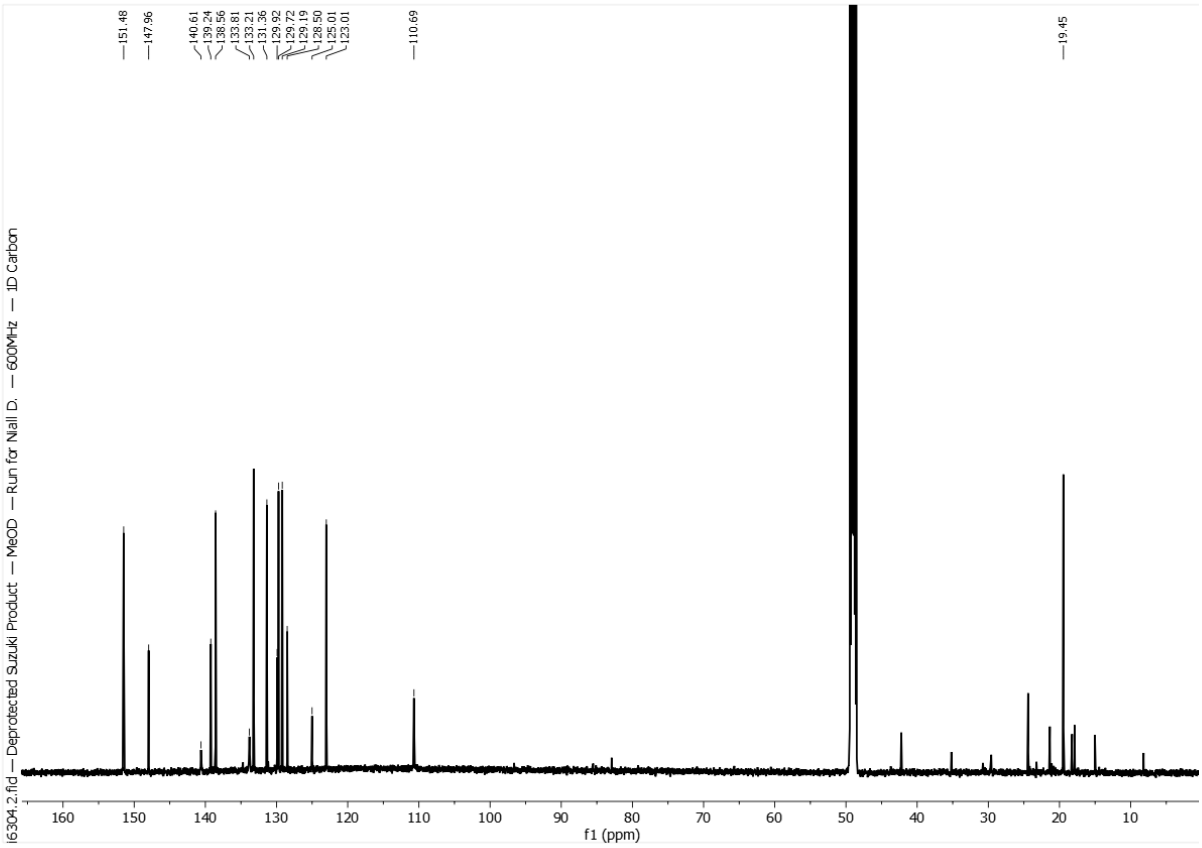

Figure S14 - Representative ^{13}C{^{1}H} NMR spectrum of compound **S1** (in Methanol-d_4).

1.4 Single crystal X-ray diffraction data

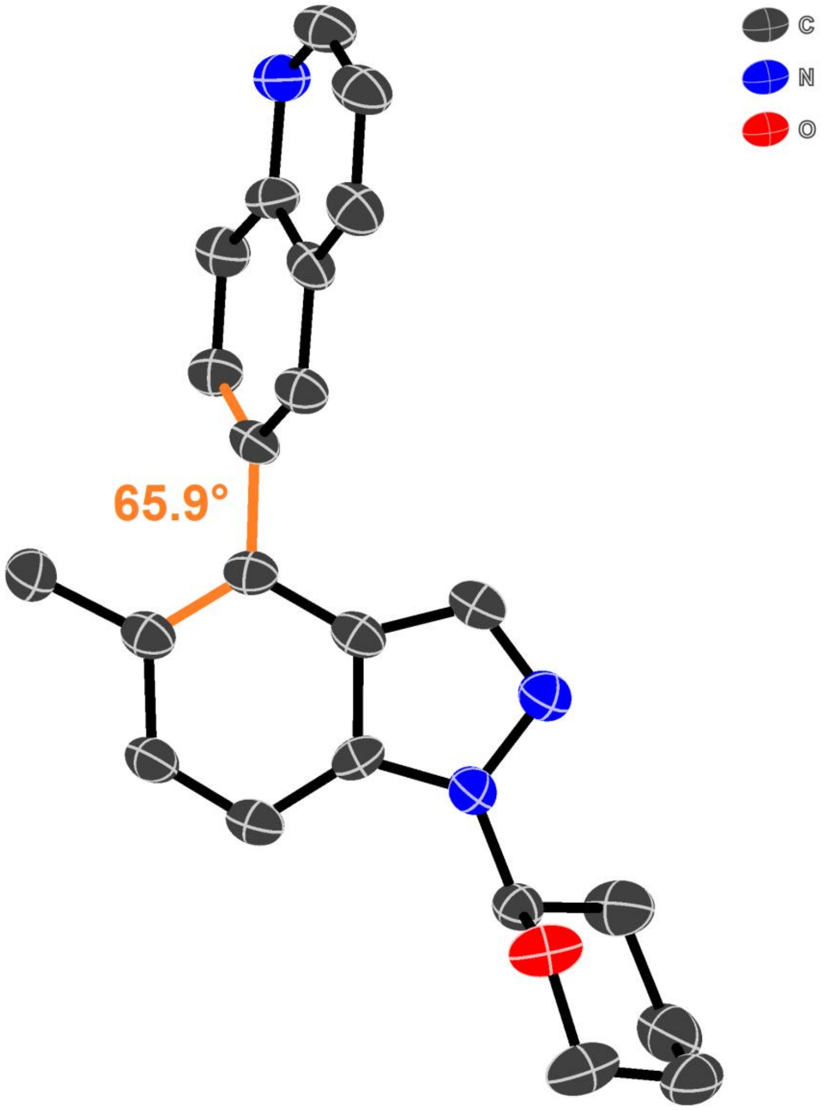

Figure S15 – Molecular structure of compound **3** (ijsf21044) by X-ray diffraction. Note the torsion angle between the indazole and quinoline ring systems (highlighted) of 65.9° indicating that there is no significant sharing of electron delocalisation between these two systems in the solid-state. This is likely as it is more energetically favourable to minimise the steric interactions between the methyl and quinolyl protons (not shown here).

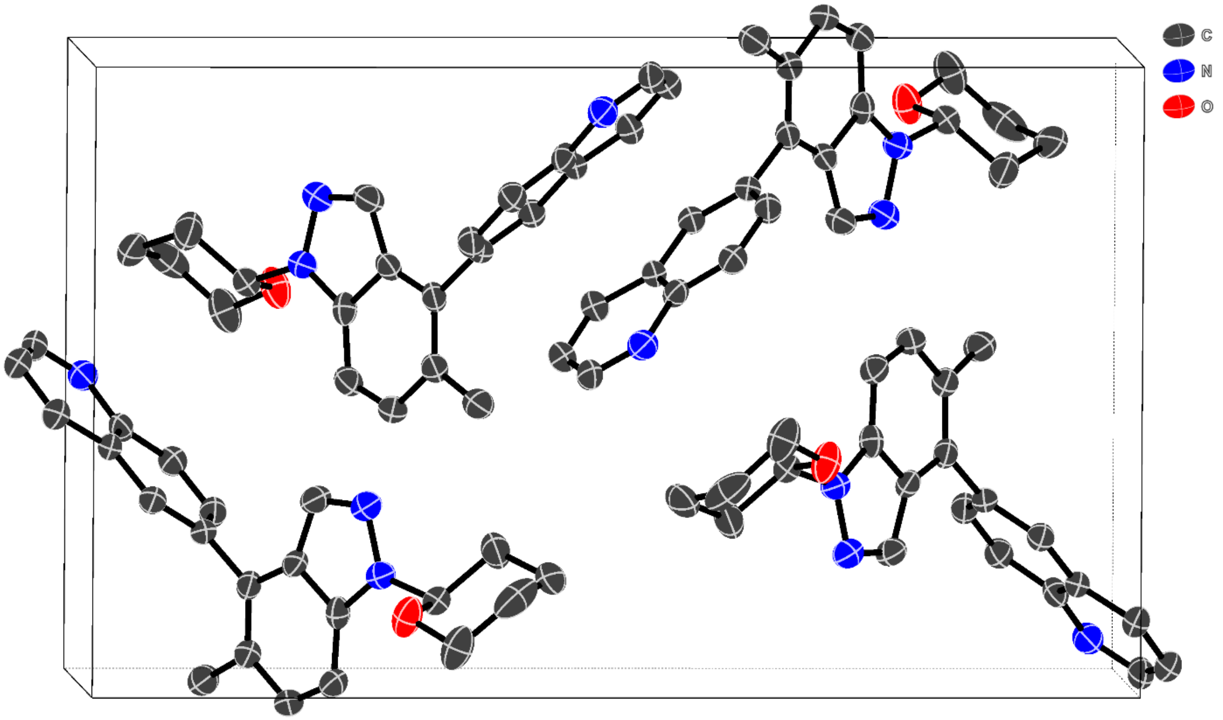

Figure S16 - Unit cell of compound **3** (ijsf21044). The predominant force determining the structure in the crystal appears to be π-π stacking interactions between the quinolyl substituents in neighbouring molecules. The unit cell consists of four molecules of **3**.

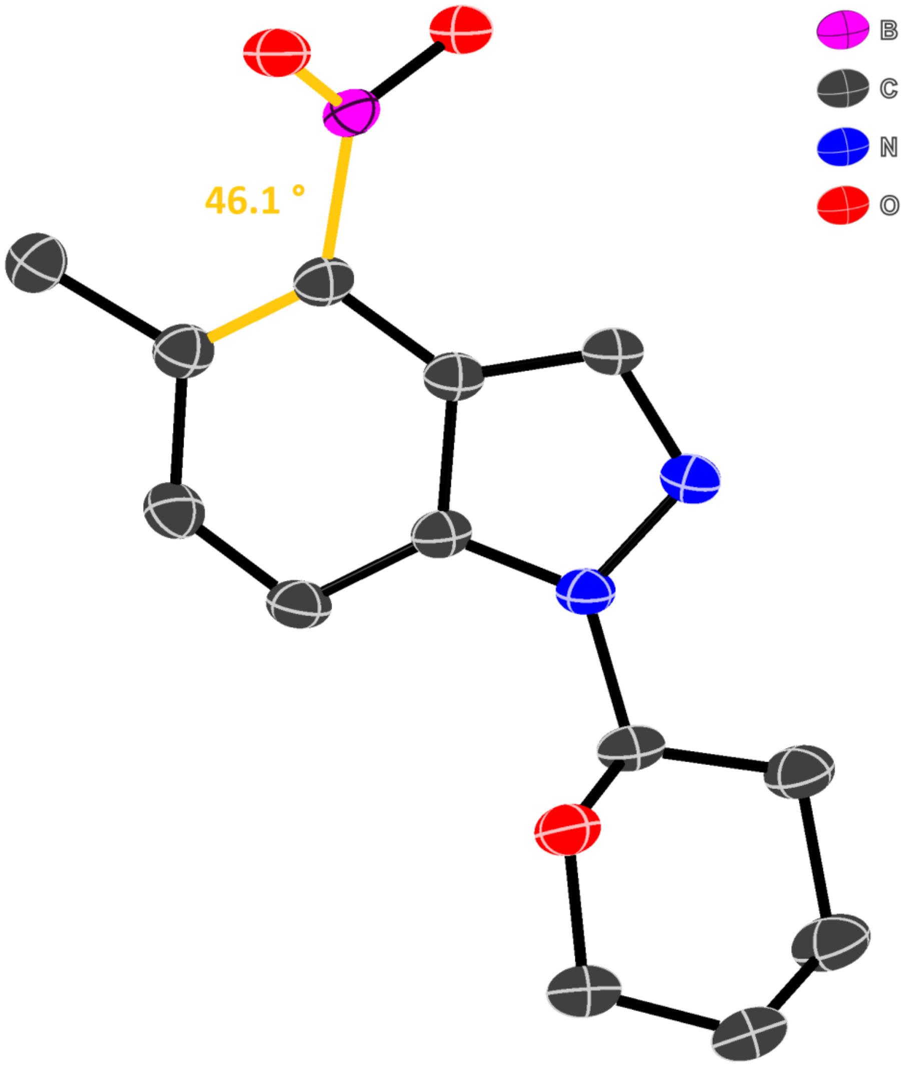

Figure S17 - Molecular structure of compound **2a** (ijsf21053) by X-ray diffraction. Bond lengths are all found to be within expected parameters.

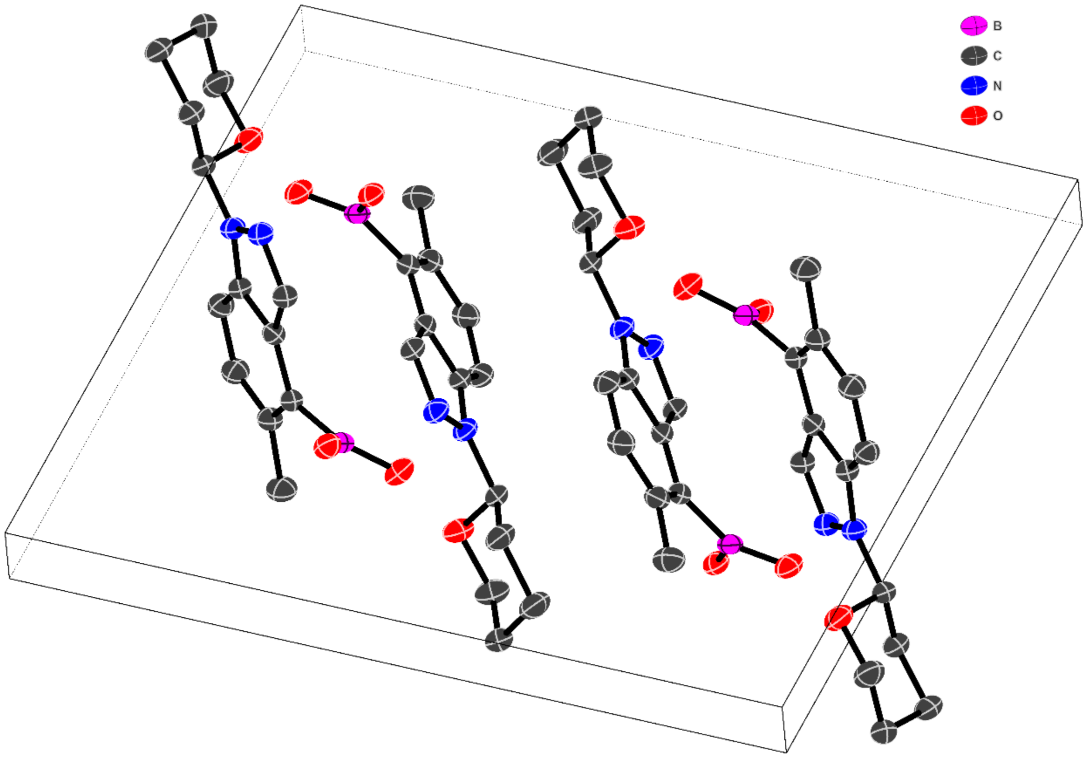

Figure S18 - The crystal contains four molecules of **2a** (ijsf21053) in the unit cell, consisting of two pairs of molecules stacked and reflected with respect to each other.

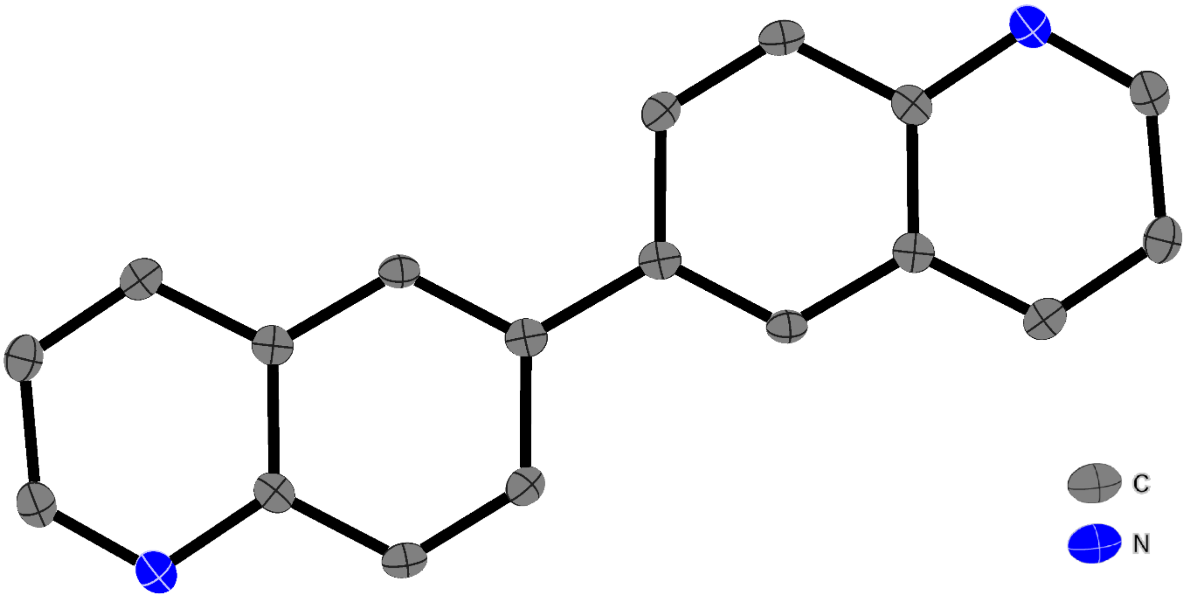

Figure S19 - Molecular structure of compound **4** (ijsf21042) by X-ray diffraction.

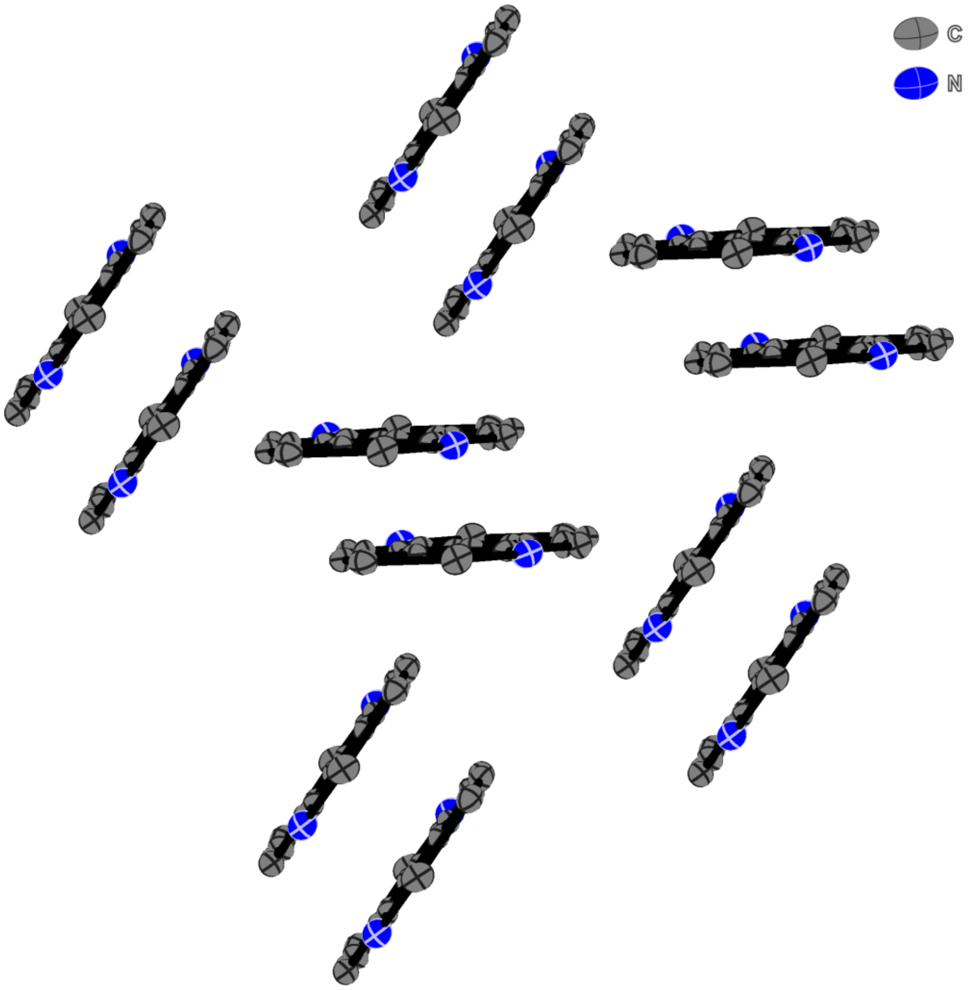

Figure S20 – View of the higher-level structure of compound **4** (ijsf21042). Both π-π and π-edge interactions are observed. Each asymmetric unit cell contains two molecules of **4**, out-of-plane with respect to each other, leading overall to pairs of planes running throughout the crystal.

Section 2: Data analysis and generation of figures

2.1 Development of the Shiny App

This shiny app (https://shiny.york.ac.uk/UOY-SMCC1/) was designed with the intention of being used as a graphical abstract – expanding on the analysis seen within the main text. All R plots found within the main text can be recreated using the shiny app. Over 500 unique plots can be visualised using this app with less than 5% of these being found in the main text. The data visualised within this shiny app contains the percentage conversions for consumed starting material(s) and formed cross-coupled products (product and side product), however when performing analysis another column of observations was added: missing product (%). Missing product is calculated by 1 – Mass balance. This additional set of measurements is available for exploration within the shiny app.

2.1.1 User Interface

When loading the shiny app, the user is greeted with a window that contains two panels: a collapsible sidebar, in which the user can adjust the graphical parameters such as graph type; and a plot window, wherein graphs appear based on the user's inputs (see Figure S21).

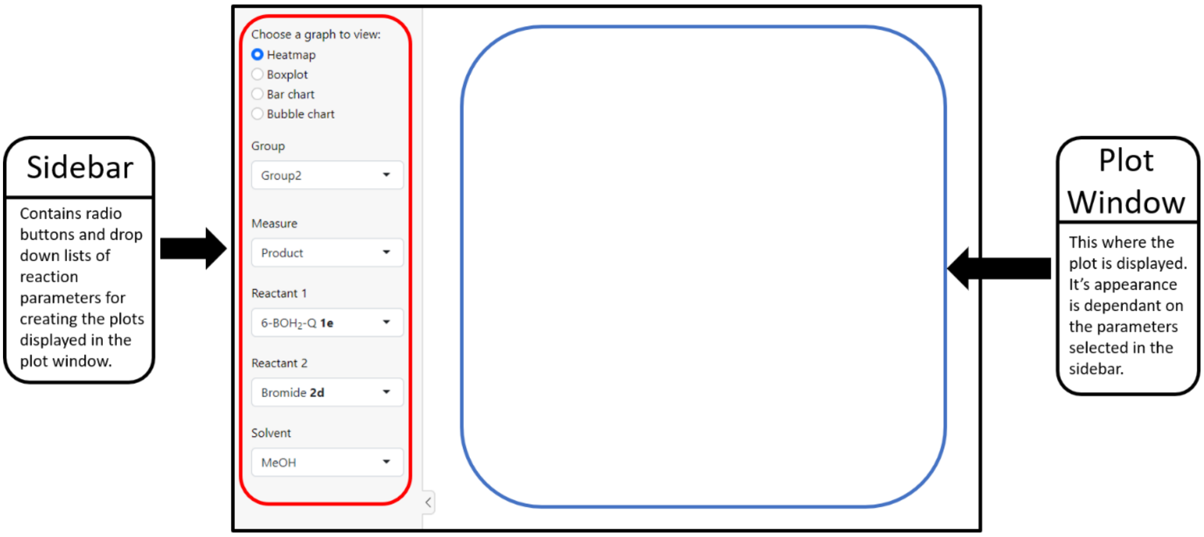

Figure S21 - Figure detailing the display of the shiny app. Sidebar on the left and plot window on the right.

The sidebar contains many parameters, the first parameter is graph choice. Graph choice allows users to select one of four graph types: heatmap, boxplot, bar chart or bubble chart. Each graph type has its own parameters that appear upon graph selection.

The 2nd parameter, group, is also constant irrespective of graph choice. Group enables the user to choose between the reaction data contained within group 1 or the reaction data in group 2 (see Figure 3 in main text).

2.1.2 Heatmap generation

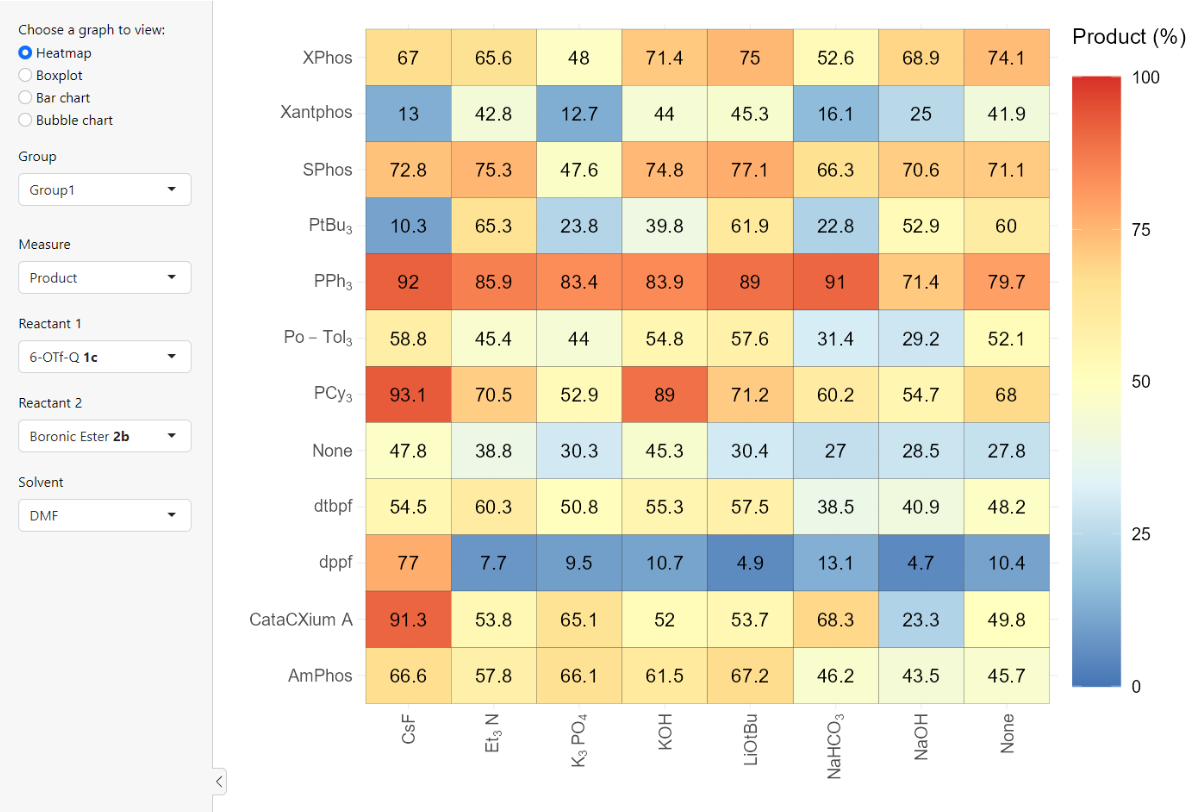

Figure S22 – A screenshot of the shiny app, showing an example heatmap created when selecting the options shown on the sidebar.

The first plot available for selection through the use of the radio buttons in the sidebar is heatmap (see Figure S22). Upon selection, the sidebar of the app updates and presents 5 drop-down boxes - the first being group as described in the shiny app section. The remaining four drop-down boxes are labelled: measure, reactant 1, reactant 2 and solvent. The measure drop-down box allows users to choose which measured variable they want to investigate, the choices are: product, side product, starting material or missing product. The 2nd drop-down box contains the choices for reactant 1: 6-Cl-Q **1a**, 6-Br-Q **1b**, 6-OTf-Q **1c**, or 6-I-Q **1d** when selecting group 1 and 6-BOH$_2$-Q **1e**, 6-Bpin-Q **1f**, or 6-BF$_3$K-Q **1g** when selecting group 2. Drop-down box reactant 2 enables users to select which reactant 2 choices to visualise, the choices are: boronic acid **2a**, boronic ester **2b**, or trifluoroborate **2c** when selecting group 1. When selecting group 2, the choice for reactant 2 is automatically fixed to bromide **2d**. The final drop-down box contains the choices of solvent and allows users to select between: DMF, MeCN, MeOH or THF.

2.1.3 Boxplot generation

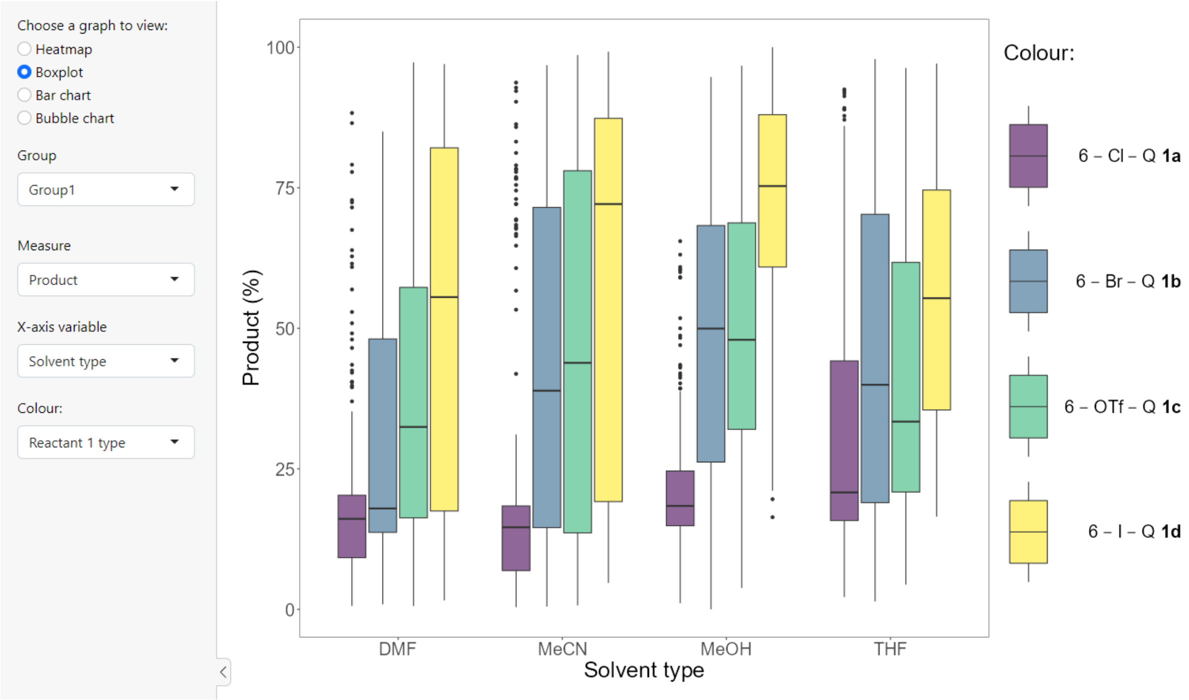

Figure S23 – A screenshot from the shiny app displaying a boxplot created when using the sidebar to select the desired parameters.

The second type of plot available in using the radio buttons is boxplot (see Figure S23). After selection, the sidebar updates to display four drop-down boxes: group, measure, x-axis variable and colour. The measure drop-down box is the same as described in the heatmap section. The x-axis variable drop-down box allows users to choose which variable they want on the x-axis, the choices are: reactant 1 type, reactant 2 type, ligand type, reagent type, or solvent type. The colour drop-down box enables the user to choose which variable to colour the plot by. The choices are the same as the choices for the x-axis variable.

2.1.4 Bar chart generation

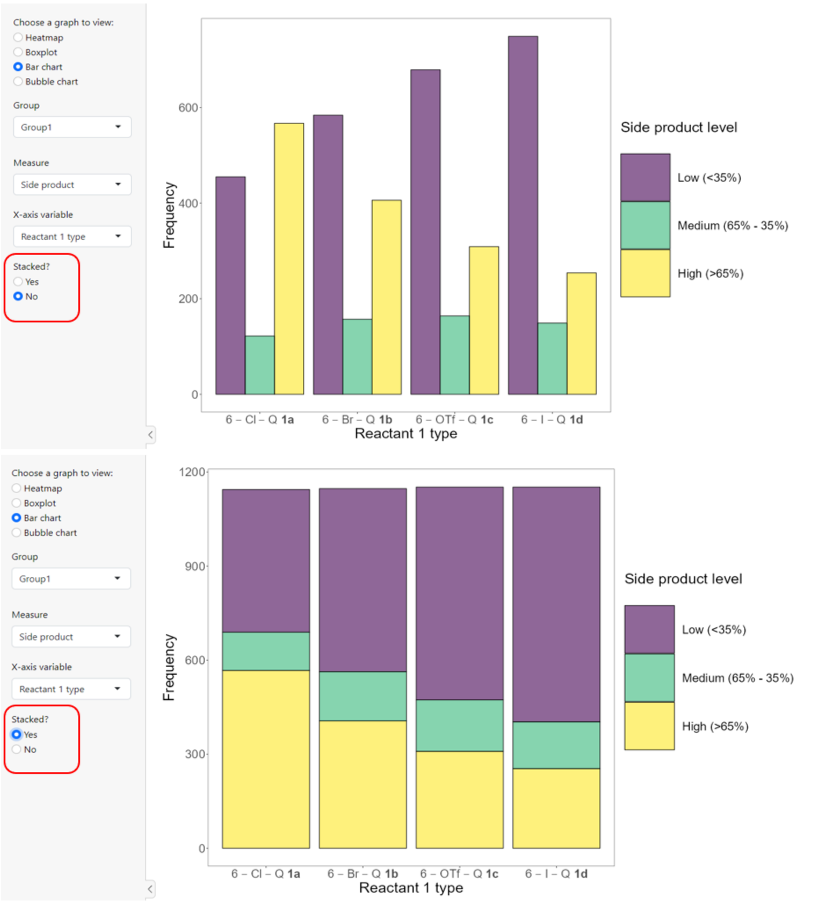

Figure S24- Two bar charts, created using the shiny app, displaying the same information in both non-stacked (top) and stacked form (bottom).

The third type of plot available for visualisation is a bar chart, when selecting this graph type, three drop-down boxes and a set of radio buttons becomes visible (see Figure S24). The set of radio buttons enables the user to swap between the unstacked and stacked version of the bar charts, both of which can be seen in Figure S24. The measure and x-axis variable drop-down boxes are the same as described in the heatmap and boxplot sections respectively.

2.1.5 Bubble chart generation

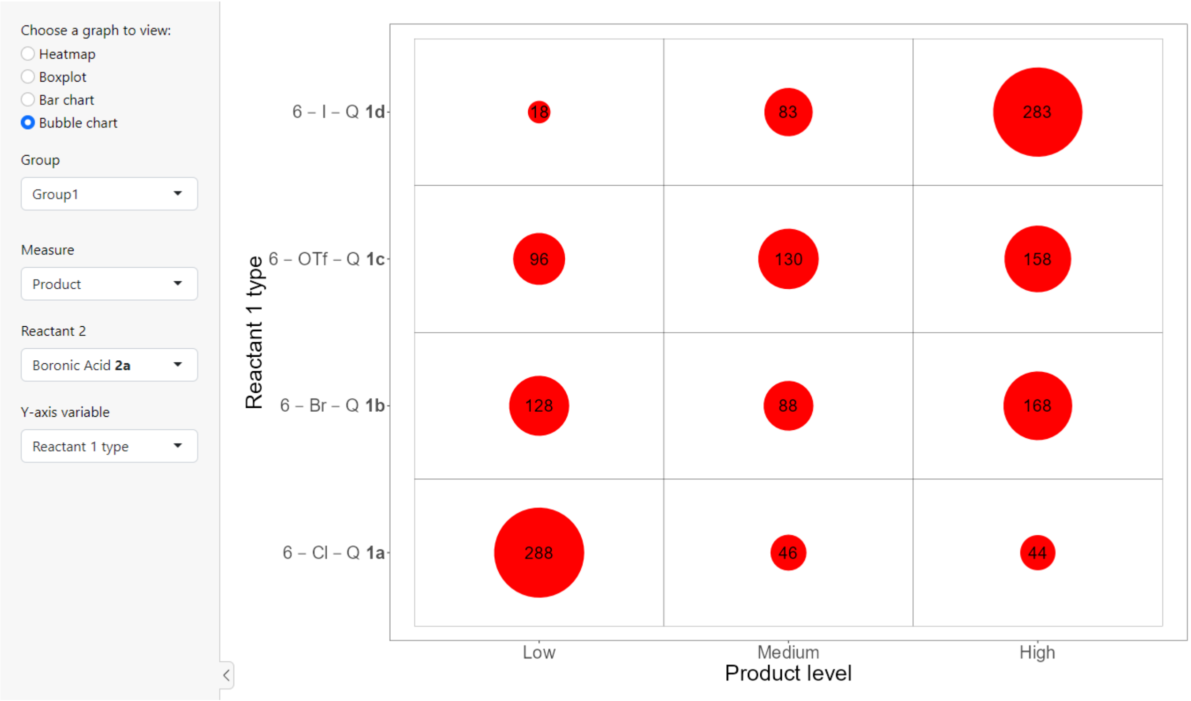

Figure S25 – A screenshot of the shiny app, displaying a bubble plot created using the parameters selected in the sidebar.

The final type of plot available for visualisation is a bubble plot (see Figure S25). There are four drop-down boxes available when selecting the bubble plot: group, measure, reactant 2, and y-axis variable. The measure variable and reactant 2 variable are as described in the heatmap section. The y-axis variable contains the same labels as the x-axis variable.

2.2 Principal component analysis

Principal component analysis (PCA) was further used to explore variance in the Group 1 dataset and any interesting trends. This tool provides additional information, as exemplified in the manuscript and in Figure S26 (below).

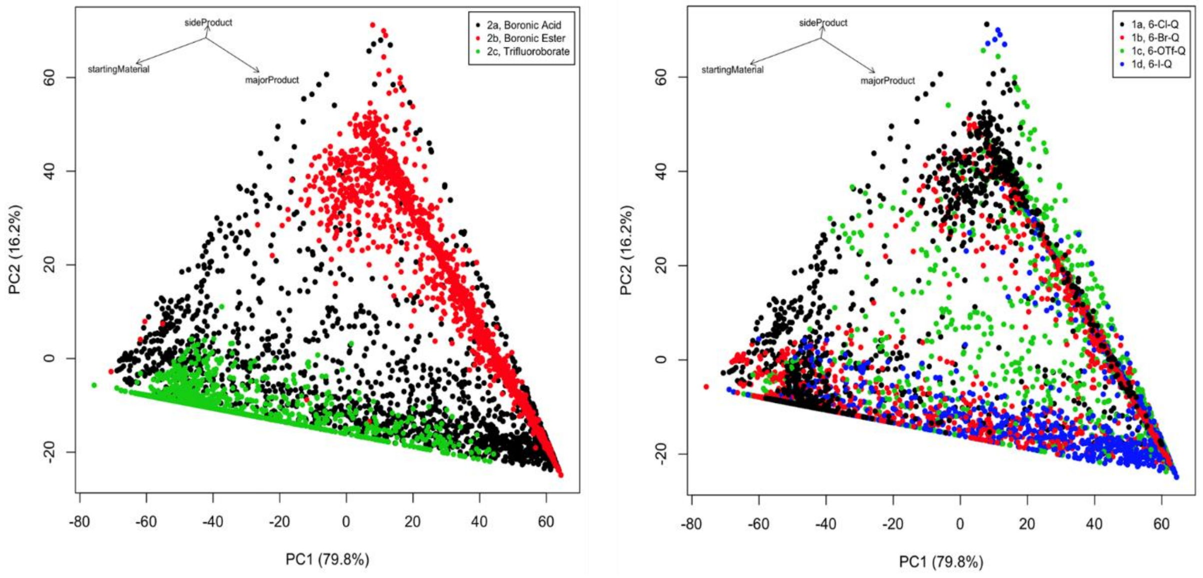

Figure S26 –Two scores plots created using principal component analysis, both plots display the same information with different variables used to colour. Left – coloured by reactant 2. Right – coloured by reactant 1.

2.3 Group 2 data analysis

Prior to analysis, across the whole dataset collected by Sach, 268 observations were removed due to inconsistencies within the dataset or due to an obvious error in the recorded variable. Of the 268 removed variables, 255 are contained within Group 2 which means that about 22% of the reactions that used bromide **2d** as starting material were removed. Additionally, 6-BOH_2-Q **1e** contains reactions that use THF V2 and MeOH V2 as solvents (along with DMF and MeCN) whereas 6-BPin-Q **1f**, and 6-BF_3K-Q **1g** use THF & MeOH. The irregularities in the data are the sole reason why Group 2 has been placed here (solvents labelled ‘V2’ is an artefact of the supplied data and the potential effects on the results is unclear).

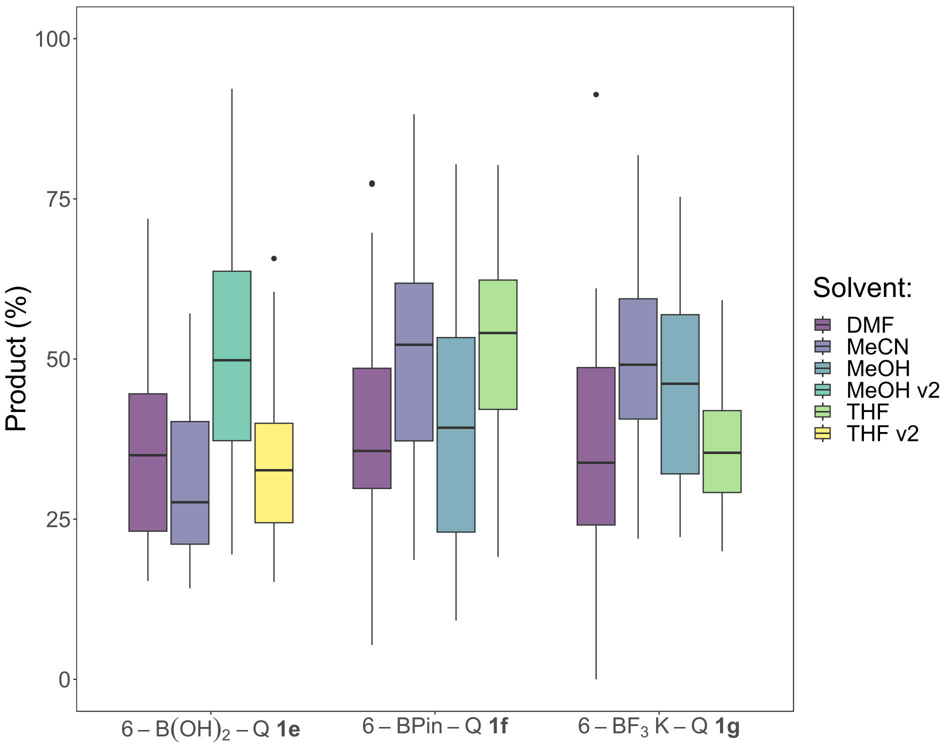

Figure S27 – Boxplot for Group 2, showing comparison of each combination of quinoline nucleophile with bromide **2d** with product on the y-axis, quinoline type on the x-axis and coloured by solvent type.

Figure S27 compares each combination of quinoline nucleophile with each solvent in Group 2 in the percentage yield of product produced. Interestingly, there doesn't appear to be an obvious choice for best nucleophile with each quinoline performing similarly. 6-BOH_2-Q **1e** performs worst for all solvents, bar MeOH – where it is best. However, it is unknown whether this has something to do with it being MeOH V2 or not. Generally, by examining this plot we can see that product yield is generally lower in Group 2 than in Group 1.

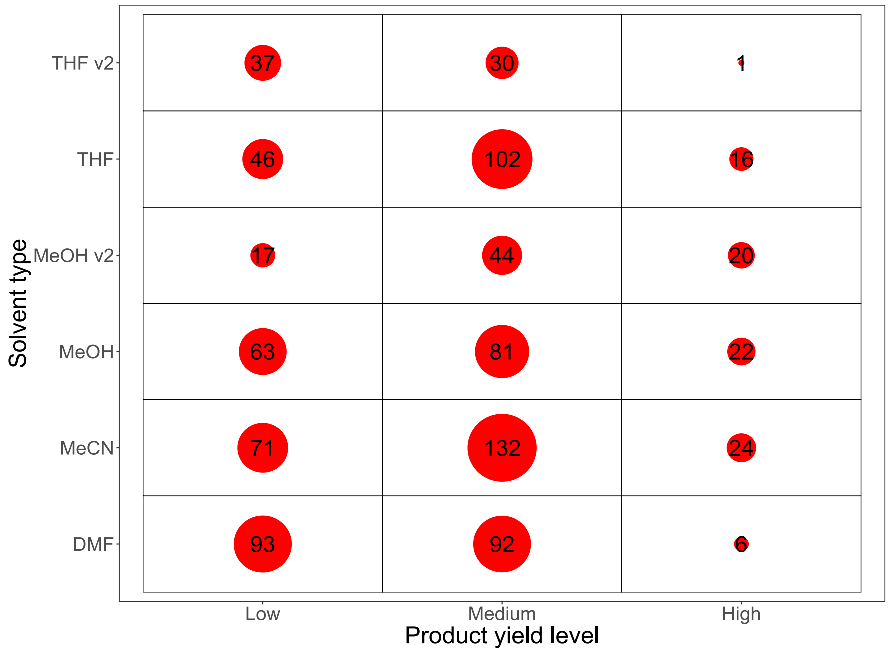

Figure S28 - Bubble count table showing the count of reactions in each product yield level by solvent type for Group 2.

In the bubble table (Figure S28), reactions lower than 35% are considered “low”, reactions in the range 35-65% are “medium” and reactions above 65% are “high”. At first glance it seemed that, MeCN was the best performing solvent as it had the most reactions in the “high” yield category. However, if we considered MeOH and MeOH V2 as the same, then methanol has almost double the number of reactions than any other solvent. Each high-yield bubble was investigated further and reactions divided up by electrophile type (**1e-1g**) with THF/THF V2 and MeOH/MeOH V2 combined:

High product reactions		Solvent					
		DMF	MeCN	MeOH	MeOH v2	THF	THF v2
Reactant1	**1e**	2	0	0	20	0	1
	**1f**	3	18	12	0	16	0
	**1g**	1	6	10	0	0	0
	Total	6	24	22	20	16	1

This once again highlights how the reactions in Group 2 perform far worse than the reactions in Group 1 with a grand total of 89 reactions producing >65% product. Figure S27 shows 2 typical heatmaps of Group 2, both showing mostly “low” yielding reactions with lots of missing data. Note that Figure S29 (bottom) has a missing row, with no recorded data for Xantphos in Group 2.

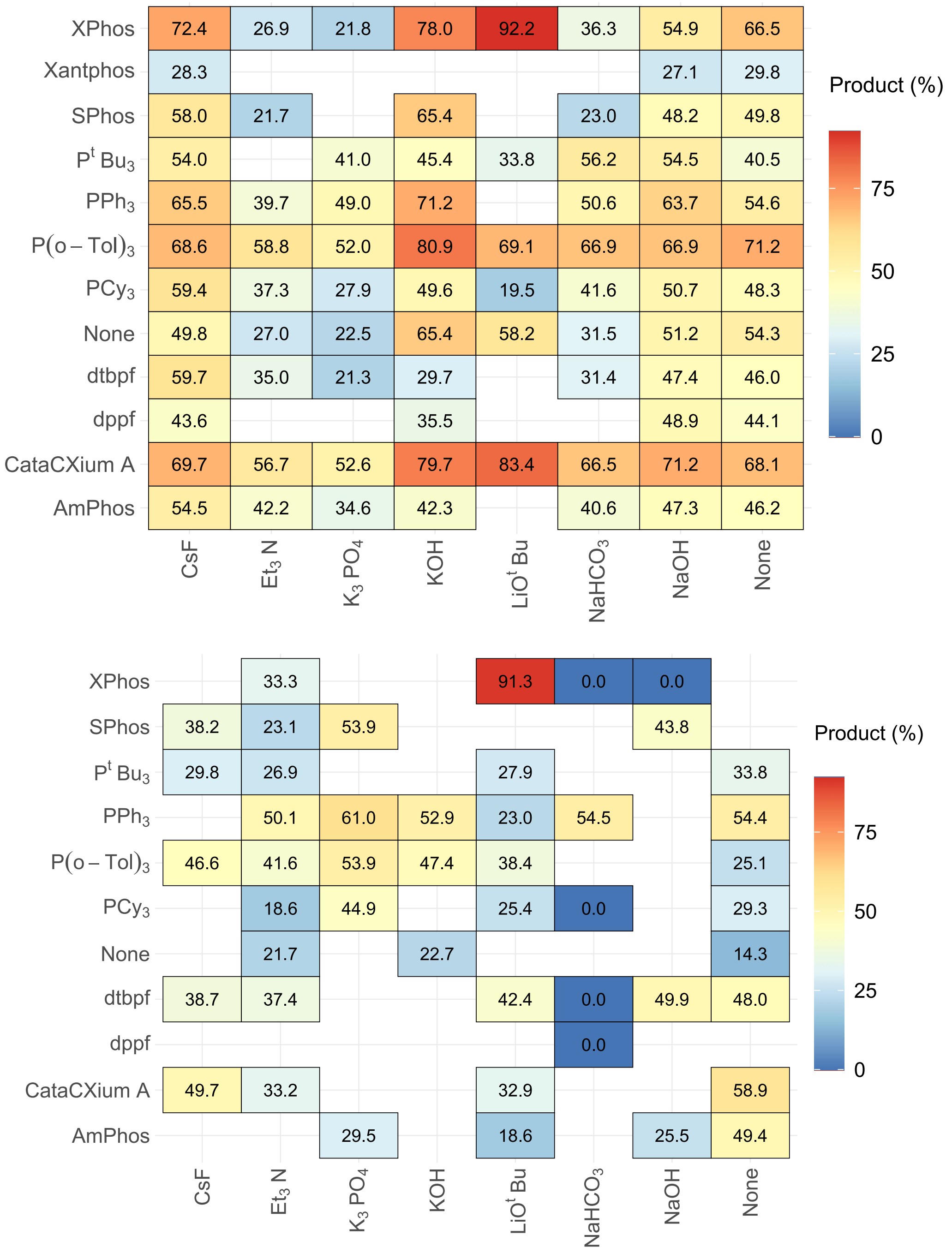

Figure S29 - Heatmap for reaction combinations using bromide **2d**. Top - 6-BOH_2-Q **1e** and MeOH. Bottom - 6-BF_3K-Q **1f** and DMF. Both plots show ligand type as the rows and base type as the columns with a heat scale ranging 0-100 where red is high, blue low and yellow in the middle

To further explore the reactions recorded in Group 2, 3 more boxplots were created. These plots are similar to the boxplot seen in Figure S27. Figure S30, shows the box plot created when comparing each quinoline nucleophile with each solvent and plotting them by side product percentage. Interestingly, all reactions examined in this space produce very little side product with only 6 reactions producing more than 25% side product.

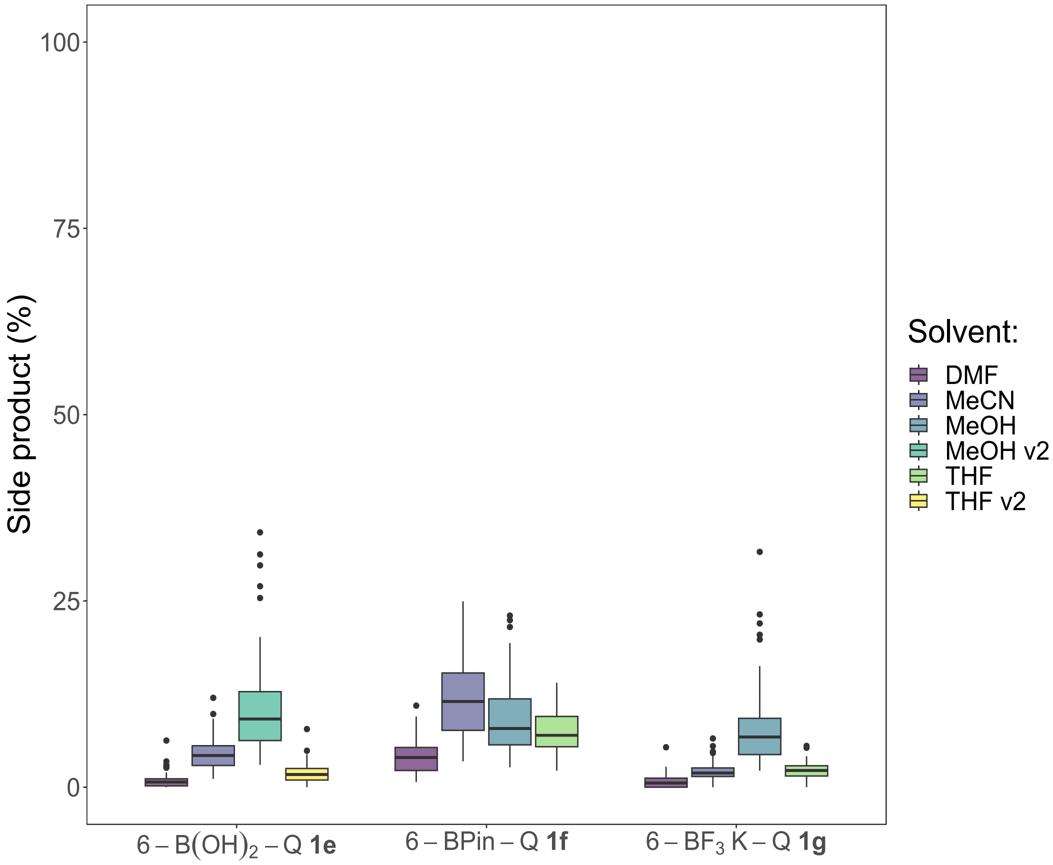

Figure S30 – Boxplot for Group 2 by side product percentage**,** showing comparison of each combination of quinoline nucleophile with bromide **2d** with side-product **2e** on the y-axis, quinoline type on the x-axis and coloured by solvent type.

Figure S31 displays boxplots showing the comparison of each quinoline nucleophile with each solvent and plotting them by starting material percentage, which in this case is the amount of bromide **2d** remaining after reaction. It is clear that the reactions in Group 2 are mostly producing starting material, or rather, that the starting materials are not reacting.

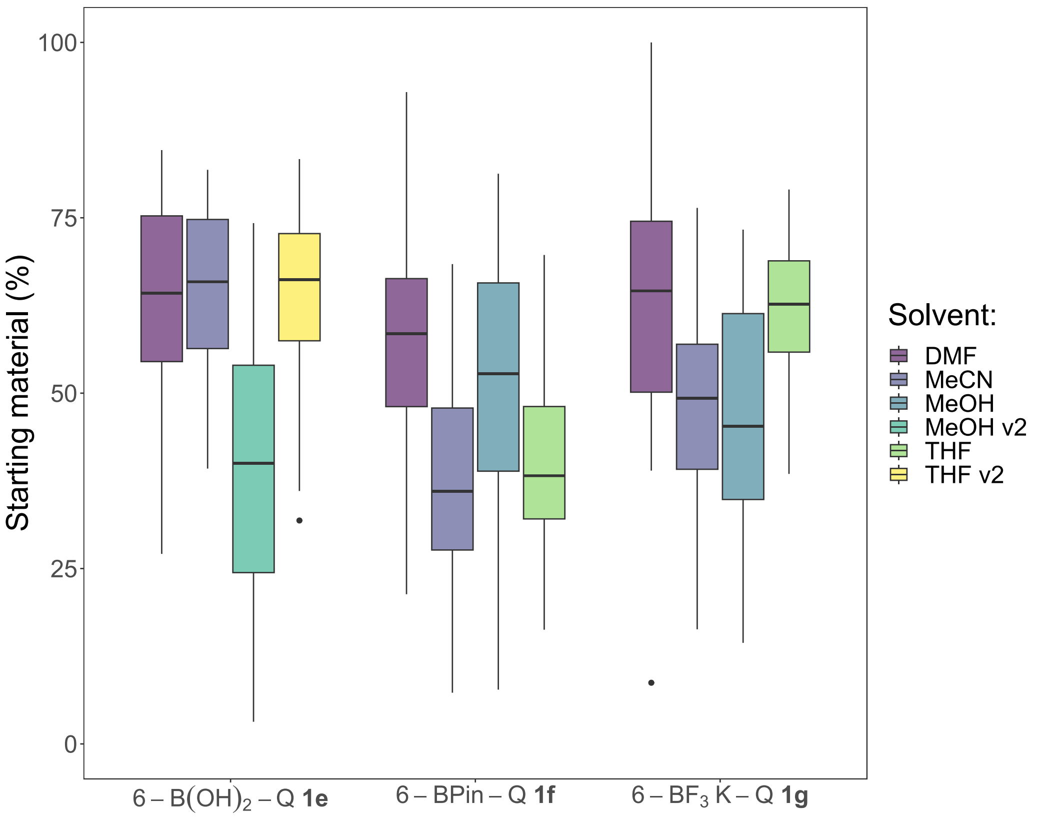

Figure S31 – Boxplot for Group 2 by starting material percentage**,** showing comparison of each combination of quinoline nucleophile with bromide **2d** with starting material on the y-axis, quinoline type on the x-axis and coloured by solvent type.

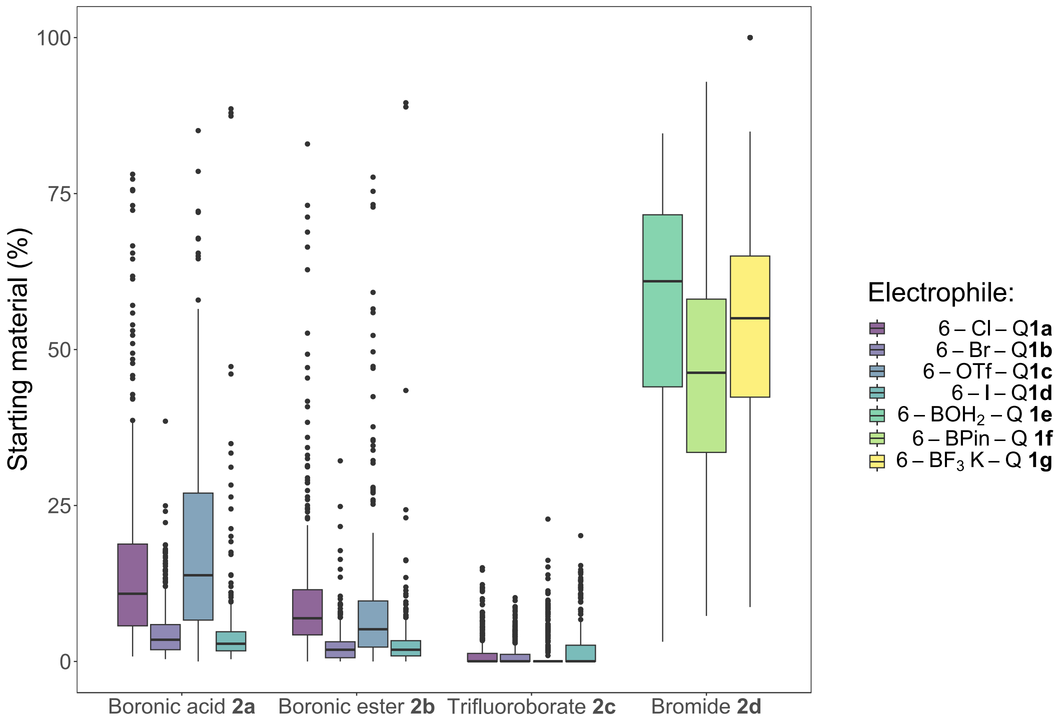

Figure S32 – Boxplot for all data both Group 1 and Group 2 with percentage starting material on the y-axis**,** showing comparison of each combination of quinoline (colour) with each indazole (x-axis).

Figure S32 shows the boxplots created when looking at all reactions in the data set and plot a boxplot for each combination of indazole and quinoline by starting material. Clearly the reactions using bromide **2d** the reactions in Group 2 contain significantly more starting material than any in Group 1.

Section 3: References

1. Sheldrick, G. M., SHELXT – Integrated space-group and crystalstructure determination, *Acta Crystallogr. A* **2015**, *71*, 3–8.
2. Sheldrick, G. M., Crystal structure refinement with SHELXL, *Acta Crystallogr. C* **2015**, *71*, 3–8.
3. Dolomanov, O. V.; Bourhis, L. J.; Gildea, R. J.; Howard, J. A. K.; Puschmann, H., OLEX2: a complete structure solution, refinement and analysis program, *J. Appl. Cryst.* **2009**, *42*, 339–341.
